# Elucidating the Role of Injury-Induced Electric Fields (EFs) in Regulating the Astrocytic Response to Injury in the Mammalian Central Nervous System

**DOI:** 10.1371/journal.pone.0142740

**Published:** 2015-11-12

**Authors:** Matthew L. Baer, Scott C. Henderson, Raymond J. Colello

**Affiliations:** Department of Anatomy & Neurobiology, School of Medicine, Virginia Commonwealth University, Richmond, Virginia, United States of America; Temple University School of Medicine, UNITED STATES

## Abstract

Injury to the vertebrate central nervous system (CNS) induces astrocytes to change their morphology, to increase their rate of proliferation, and to display directional migration to the injury site, all to facilitate repair. These astrocytic responses to injury occur in a clear temporal sequence and, by their intensity and duration, can have both beneficial and detrimental effects on the repair of damaged CNS tissue. Studies on highly regenerative tissues in non-mammalian vertebrates have demonstrated that the intensity of direct-current extracellular electric fields (EFs) at the injury site, which are 50–100 fold greater than in uninjured tissue, represent a potent signal to drive tissue repair. In contrast, a 10-fold EF increase has been measured in many injured mammalian tissues where limited regeneration occurs. As the astrocytic response to CNS injury is crucial to the reparative outcome, we exposed purified rat cortical astrocytes to EF intensities associated with intact and injured mammalian tissues, as well as to those EF intensities measured in regenerating non-mammalian vertebrate tissues, to determine whether EFs may contribute to the astrocytic injury response. Astrocytes exposed to EF intensities associated with uninjured tissue showed little change in their cellular behavior. However, astrocytes exposed to EF intensities associated with injured tissue showed a dramatic increase in migration and proliferation. At EF intensities associated with regenerating non-mammalian vertebrate tissues, these cellular responses were even more robust and included morphological changes consistent with a regenerative phenotype. These findings suggest that endogenous EFs may be a crucial signal for regulating the astrocytic response to injury and that their manipulation may be a novel target for facilitating CNS repair.

## Introduction

The regenerative potential of the vertebrate central nervous system (CNS) is, in large part, determined by the astrocytic response to the injury [1,2]. Common among all vertebrates studied, astrocytes begin migrating toward the lesion within hours of injury [3,4], and they proliferate beginning within 24 hours and peaking after 48 hours [4–10]. This initial response is necessary to reestablish the blood-brain barrier (BBB), and impairing either migration or proliferation allows the lesion to expand into the surrounding healthy tissue [11–15]. Subsequently, astrocytes in mammals decrease their rate of proliferation towards baseline levels within 72 hours of the lesion [5,6,8,15], they increase their expression of the intermediate filaments glial fibrillary acidic protein (GFAP) and vimentin over the first 1–5 days after the injury [5,10,13,16], and they release molecules that limit spontaneous axon sprouting and inhibit regeneration [1,17–21]. In contrast, astrocytes in non-mammalian vertebrates sustain an increased rate of proliferation for over a week post injury [7,22], they decrease their expression of GFAP relative to astrocytes in the uninjured CNS and instead increase their expression of nestin [7], they migrate into the injury site and form a cellular bridge across the lesion [4,23,24], and they assume a bipolar morphology with highly-aligned cellular processes that guide sprouting axons and facilitate robust regeneration [4,25]. The fact that the initial astrocytic response to injury is highly conserved among vertebrates suggests that the stimulus initiating this response may be similarly conserved, but that this stimulus does not reach the threshold in mammals that is necessary to sustain those astrocytic behaviors that facilitate robust regeneration in non-mammalian vertebrates [2]. If this is the case, this stimulus would be an ideal therapeutic target to modify the mammalian astrocytic response towards that seen in successfully regenerating animals and thus enhance regeneration in the mammalian CNS.

Direct-current extracellular electric fields (EFs), which are voltage gradients within tissues produced by spatial variations in epithelial cell ion pump activity [26–30], may be the stimulus that directs astrocyte behavior after injury in the vertebrate CNS. EFs have been shown to have an intensity-dependent effect that directly induces cellular behaviors–including migration [31–37], proliferation [38–42], differentiation [33,43,44], and morphology [37,45–49]–among a variety of ectodermally- and mesodermally-derived cell types [26–28,50]. EFs, which are typically less than 10 mV/mm in intact tissues [51–53], increase substantially upon injury. In non-mammalian vertebrates, a 50- to 100-fold increase in EFs has been measured upon injury in the skin [54–56], bone [57], cornea [58,59], lens [60–62], spinal cord [63], tail [64,65], and limb [66–71], and this EF increase has been shown to be both necessary and sufficient to induce regeneration [66,72–83]. In mammals, EFs only increase approximately 10-fold upon injury–including in the skin [29,52,53,84,85], respiratory epithelium [86], cornea [30,40,59], lens [87,88], bone [57,89,90], and finger amputation [91]–where injury resolution occurs by scar formation. Interestingly, increasing the EF intensity towards levels found in non-mammalian vertebrates promotes regeneration in these mammalian tissues [40,80,92–94]. Within the mammalian CNS, EFs of 3.5–5.0 mV/mm have been recorded *ex vivo* in the rostral migratory stream [51], slice culture induces a 10-fold EF increase to 31.8 ± 4.5 mV/mm in the subventricular zone [95], and current density–which is directly proportional to the EF [96]–increases 10-fold upon spinal cord injury [97,98]. If endogenous EFs in the CNS have an intensity-dependent effect on regeneration that is analogous to their established role in peripheral tissues in both mammalian and non-mammalian vertebrates [99–102], then EFs may be the stimulus that regulates the astrocytic response to injury and therefore determines the regenerative potential of the CNS.

EF intensities associated with regenerating tissues have been shown to promote a regenerative phenotype in neurons by enhancing neurite outgrowth *in vitro* and axon sprouting *in vivo* [47,49,74,103–114], but the physiologic relevance of this neuronal response is unknown as astrocytes determine whether these sprouting axons can regenerate past a lesion site. Previous research by our lab and others has demonstrated that EF intensities associated with regenerating tissues induce process alignment in mammalian astrocytes similar to that facilitating regeneration in non-mammalian vertebrates [112,115,116]. Moreover, these EF-exposed astrocytes facilitate robust neurite outgrowth [115]. However, as these studies were limited in the EF intensities evaluated, the exposure times tested, and the cell behaviors observed, it remains unclear to what extent EFs can modulate mammalian astrocyte behavior. In the current study, we tested the hypotheses that physiologic EFs produced by the injured mammalian CNS are sufficient to induce behaviors associated with the astrocytic injury response, and that increasing these EFs to levels found in regenerating non-mammalian vertebrates will modify the behavioral response towards one associated with a regenerative phenotype *in vivo*. Our findings suggest that injury-induced EFs are an important stimulus for the astrocytic response to injury, and that the EF intensity determines whether the induced behaviors reflect a phenotype associated with glial scar formation or with regeneration. Thus, EFs may represent a novel target to enhance the regenerative potential in the mammalian CNS.

## Methods

### Cell Source and Culture Methods

**Fig 1:**
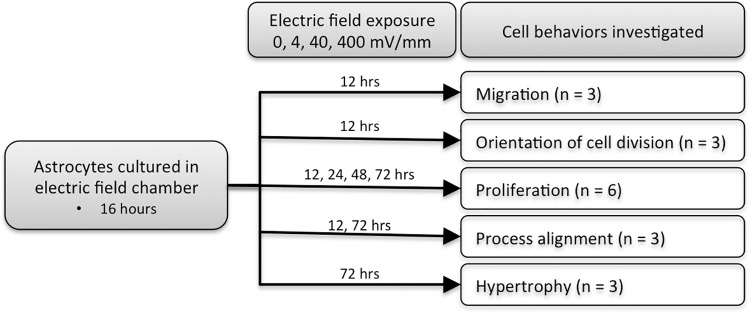
Flow chart outlining study methodology. Astrocytes were exposed to 0, 4, 40, or 400 mV/mm. The time points used for each behavioral endpoint are indicated. The number of cultures exposed to each EF intensity at each time point is indicated for each behavioral endpoint.

Rat cortical astrocytes harvested from P2 cerebral cortex were purchased from ScienCell (cat # R1800). Cultures have greater than 99% purity as determined with GFAP immunolabeling by ScienCell. All of the astrocytes used for these experiments came from the first five passages after the initial thaw. Astrocyte cultures were maintained according to the protocol recommended by ScienCell. Briefly, astrocytes were thawed into poly-L-lysine (ScienCell # 0413) coated T75 culture flasks containing astrocyte media (pH 7.4; ScienCell AM-a 1831) supplemented with 2% fetal bovine serum (ScienCell # 0010) and 1% penicillin/streptomycin (ScienCell # 0503). Cultures were maintained in a humidified 37°C incubator with a 5% CO_2_ atmosphere, and culture media was changed every 2–3 days. Once the cultures reached confluence, approximately 5,000 astrocytes were sub-cultured into each EF chamber (see description below) for migration, proliferation, and morphology experiments (Fig 1 illustrates the time points used for each of the behavioral assays). For proliferation assays, 5-bromo-2’-deoxyuridine (BrdU; Invitrogen, cat # 00–0103 diluted 1:100 in astrocyte media) was added to the culture for the last 6 hours of the EF exposure. Each experiment was replicated at least three times using cortical astrocytes derived from different animals (different lot numbers of astrocytes were purchased from ScienCell), with all cells used in a given experiment being sister cultures derived from the same passage.

### Electric Field Chamber Design and Application

**Fig 2:**
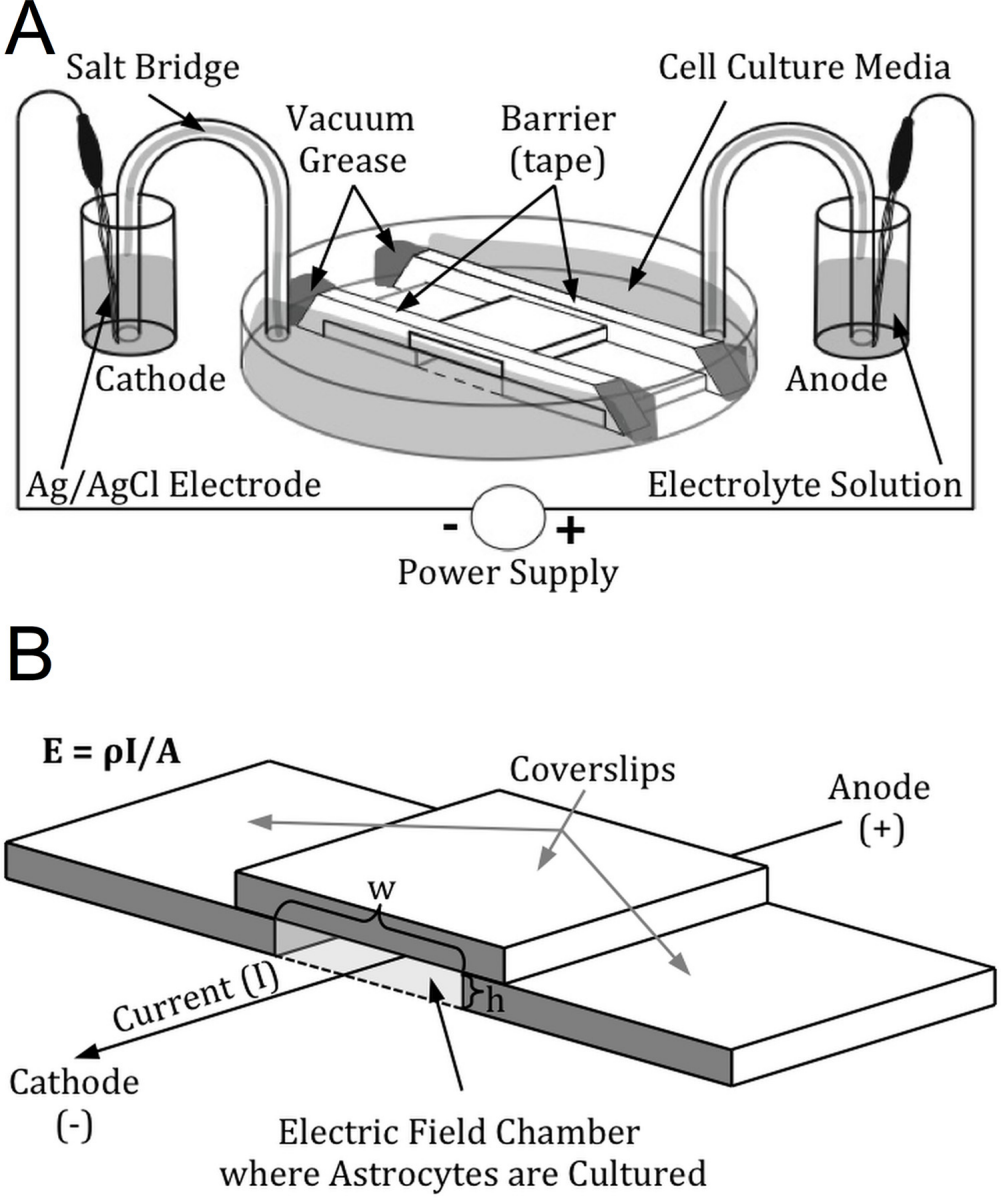
Electric field chamber. (A) An illustration of the electric field chamber, showing how it is connected to the circuit that creates the EF. The power supply drives a redox reaction at each of the electrodes, converting the electrical current into an ionic current through the electric field chamber with cations moving towards the cathode (negatively-charged electrode) and anions moving towards the anode (positively-charged electrode). (B) Enlarged view of the EF chamber, illustrating how specific EF are calculated and applied. EF magnitude is calculated with the formula *E = ρI/A*. Varying the cross-sectional area of the EF chamber (using coverslips of different thicknesses (h) and changing the distance between them (w)) and the magnitude of the applied currents creates different EF strengths.

Electric field chambers were constructed in a similar manner to those described by Babona-Filipos et al (2012) and Song et al (2007) [82,117], with some modifications as described below (Fig 2). EF chambers were constructed using 50 x 7 mm glass-bottom petri dishes (Ted Pella, #14027) (Fig 2A). Acid-washed 22 mm x 22 mm—1.5 glass coverslips (average thickness 0.17 mm) were cut into two equal rectangles (11 mm x 22 mm) using a diamond knife. These coverslips were adhered to the bottom of the petri dish with hot dental wax to create a 10 mm x 22 mm x 0.17 mm central chamber. EF chambers were then sterilized under a UV light for at least 30 minutes, coated with fibronectin (ScienCell # 8248) for 30 minutes (10 μg/mL in 0.1M phosphate-buffered saline, PBS), rinsed twice with deionized water, and then allowed to dry. 5000 astrocytes were seeded onto these chambers and allowed to adhere to the dish overnight (at least 16 hours). At the start of the experiment, a 22 mm x 22 mm—1.5 glass coverslip was used to create a roof for the EF chamber by using sterilized silicone vacuum grease (Dow corning # 1966898–0712) to seal it to the cut coverslip spacers on either side of the cell culture lane. Double-sided tape placed over the coverslip roof on either end of the lane created wells for additional culture media; the junction between the double-sided tape and the edges of the petri dish were made water-tight by sealing the gaps with additional silicone vacuum grease to prevent any media from leaking around the barriers. It is important that a good seal is maintained so that the only aqueous connection between the wells on either end of the culture dish is through the central trough containing the cells; otherwise, the applied current may leak around the area where the cells are cultured, which would cause the actual applied electric field to be less than the calculated EF.

Constant-current EFs were applied to the cells by connecting the EF chamber to a power supply; the position of the anode and cathode determine the orientation of the electric field and are indicated in figures as either A/C or +/-, respectively. Two constant current power supplies were used, including Bio-Rad Power Packs 1000, and Stoelting Precision Current Source 51413, in order to provide the full range of current needed for these experiments. The power-packs were connected to Ag/AgCl electrodes (made by washing silver wire, Alfa Aesar 45852, in an HCl/HNO_3_ solution for 15 seconds and then rinsing it in dH_2_O), which were immersed in 50 mL flasks containing Steinberg Solution (54 mM NaCl, 0.7 mM KCl, 1.6 mM MgSO_4_, 0.4 mM Ca(NO_3_)_2_, 1.4 mM Tris); these were connected to the EF chambers through salt bridges made from a 2% agarose solution suspended in 2 mL plastic pipets that were bent into a U shape. For EF experiments lasting longer than 24 hours, culture media and salt bridges were completely replaced every 24 hours.

The magnitude of the electric field strength (*E*) (units: millivolts per millimeter, mV/mm) is calculated according to the formula *E* = *ρI*/*A*, where the resistivity of the media (*ρ*) was measured as 700 Ωmm, the applied current (*I*) is specified by the power supply, and the cross-sectional area of the EF chamber (*A*) is calculated in mm^2^ (as a function of the thickness of the coverslip spacers (*h*) and the width of the cell culture chamber as defined by the distance between the spacers (*w*); Fig 2B). EF strength is controlled by specifying the applied current and by changing the cross-sectional area of the cell culture chamber (by using spacers of different thickness, or by changing the width of the cell culture chamber). We applied an EF of 0, 4, 40, or 400 mV/mm throughout the entire experiment by delivering a constant current of either 0, 10, 100, or 1000 μA. Ammeters in series with the EF chambers were used to monitor the value of the applied current throughout the experiments.

### Time-lapse imaging

Electric field chambers were placed on a Zeiss AxioObserver Z1 inverted microscope (Carl Zeiss, Jena Germany) equipped with a fully automated and programmable Mährhäuser scanning stage, an Axiocam MRm camera, and a stage incubator system that regulates temperature, oxygen, and carbon dioxide throughout the experiment. The cell culture chamber was placed on the stage and the incubation chamber was maintained with a humidified 5% CO_2_ environment at 37°C. A 20x 0.8 NA Plan-Apochromat objective lens was used to acquire images with differential interference contrast (DIC) optics every 3 minutes for the duration of the experiment, and these images were subsequently stitched together into time-lapse videos. Image acquisition was automated using the Zen Blue (2012, version 1.1.2.0) software package. We began imaging the first time-lapse video approximately 16 hours after the cells were seeded into the EF chambers. Cells were imaged for at least 30 minutes prior to the start of the EF exposure to establish baseline cellular behavior, and then for at least 12 hours after EF onset. As only one dish could be imaged at a time and each experiment ran for 15 hours, the last group that was imaged had been growing in the EF chamber for up to 36 hours longer than the first culture had been. To control for potential sequence effects (i.e. cells changing their responsiveness to EF exposure as a function of the length of time that has elapsed since they were sub-cultured into the EF chamber), we specified a different order of exposure to each of the EF strengths between experiments. We also directly tested whether the delay between sub-culturing the cells and beginning the EF exposure had any effect on the cellular response to the EF by exposing sister cultures to 40 mV/mm for 12 hours beginning either 16 or 48 hours after sub-culturing into the EF chamber. We found no evidence to suggest that this delay affected the response to the EF exposure, so data were pooled across experiments for the analysis.

### Migration Analysis

To analyze astrocyte migration, time-lapse videos were imported into ImageJ and analyzed using the plugin MTrackJ [118]. To track each cell, the point corresponding to the center of each cell’s nucleus was manually selected in every 5^th^ frame (15 minutes) throughout the 12-hour experiment, and these positions were used to calculate the magnitude of the velocity (i.e. speed) and the direction of migration at each 15-minute interval. At least 8 representative fields of view were acquired for each experiment, and 4–7 cells with representative morphologies were selected from each field of view and tracked every 15 minutes; when a cell moved out of the field of view, we began to track a new cell at the second-to-last time point before the first cell moved out of the field of view. Thus, a minimum of 30 cells were tracked for each time point for each experiment (thus allowing the use of parametric statistical inference regardless of potential skew of the population distributions for cell speed), resulting in an overall n ≥ 90 cells for each EF strength at each time point once data for all 3 experiments were pooled. All data on cell tracking produced by MTrackJ for the migration analysis were compiled in Microsoft Excel 2011, saved as comma separated values files, and then imported into the statistical program R [119]. All data analyses were performed using R (including the packages Circular [120], Ggplot2 [121], Pastecs [122], Reshape [123], and Multcomp [124]), with RStudio [125]. The vector representing each cell’s velocity was broken down into the speed and direction components, and each component was analyzed individually. Mean cell speed was compared at each time point for statistical significance using a 1-way ANOVA with Tukey HSD post-hoc tests, with an overall threshold for significance at each time point of *p* = 0.05. The ability of each EF to induce directional cell movement was assessed at each time point using Rayleigh’s test, using a *p-*value = .05 with a Bonferroni correction for the number of comparisons (196 comparisons: 4 EF levels; 49 time points). For those EF strengths and time points where there was directional migration we measured the mean direction of alignment (μ ± SEM) and the dispersion of direction about the mean angle with the concentration parameter (κ ± SEM). The precision of migration was compared at each time point between EF-exposure groups that demonstrated directional migration using the equal kappa test, which evaluates the null hypothesis that the concentration parameter is equal for each of the two groups against the alternative hypothesis that the concentration parameters differ between the two groups using an overall threshold for significance at each time point of *p* = 0.05.

### Analysis of Electric Field Effects on the Orientation of the Axis of Cell Division

To determine whether the EF exposure aligned the axis of cell division, each mitotic event in the time-lapse live cell videos was identified. The orientation of the axis of cell division was measured by drawing a line between the center of each of the daughter nuclei in the first frame where the two daughter nuclei are distinctly identifiable. Image analysis was completed using the program Fiji [126]. The angle of this line relative to the axis of the EF was measured. Alignment of the mitotic axis was determined for each EF strength using a Rayleigh’s test, with *p* < 0.01 as the threshold for determining significance (*p* < 0.01 was chosen as a conservative adjustment for multiple comparisons based on a nominal *p* < 0.05 for 4 different groups). If the sample showed statistically significant alignment, the mean angle, concentration parameter (κ), and angular standard deviation are reported.

### Immunocytochemistry

Cells from each independently-derived population were immunolabeled for GFAP, vimentin, and nestin to determine the purity and maturational state of the astrocyte population. To assess EF-induced effects on astrocyte morphology and hypertrophy, astrocytes were exposed to an EF of either 0, 4, 40, or 400 mV/mm for either 12 or 72 hours. For immunolabeling astrocytes after EF exposure, cells were rinsed with ice-cold 0.1M PBS (pH 7.4) and then fixed with 4% paraformaldehyde in 0.1M PBS for at least 12 hours. Cells were washed 3 times with 0.1M PBS, blocked and permeabilized (4% normal goat serum, 0.5% bovine serum albumin, and 1% Triton X100 in 0.1M PBS) for 30 minutes at 25°C, and then incubated with the primary antibodies diluted in this blocking solution either for 2 hours at 25°C or overnight at 4°C. Cells were then washed 3 times with 0.1M PBS, incubated with fluorescent-tagged secondary antibodies (diluted in 0.1M PBS) for 2 hours, counter-stained with DAPI (NucBlue Fixed Cell ReadyProbes kit, diluted per manufacturer’s instruction; Molecular Probes # R37606), and mounted under glass coverslips with Vectashield (Vector Labs # H-1000). For cells immunolabeled for BrdU, an additional acid wash series was used to expose the BrdU for antibody binding prior to the start of the immunolabeling protocol: cell DNA was denatured for 10 minutes in 1N HCl on ice, 10 minutes in 2N HCl at 25°C, and 20 minutes in 2N HCl at 37°C, and then neutralized with 0.1M borate buffer for 10 minutes at 25°C. Primary antibodies used in the immunocytochemistry studies included the following: mouse IgG_1k_ anti-BrdU (1:1,000; Dako # M0744), polyclonal rabbit anti-GFAP (1:5,000; Dako # Z0334), polyclonal chicken IgY anti-vimentin (1:1,000; Millipore # AB5733), mouse IgG_1_ anti-nestin (1:1,000; clone rat-401, Millipore # MAB353). Secondary antibodies used were Goat IgG anti-rabbit Alexa-488 (Molecular Probes # A-11008), Goat IgG anti-chicken IgG Alexa-568 (compatible with chicken IgY primary antibody; Molecular Probes # A-11041), and Goat IgG anti-mouse Alexa-647 (Molecular Probes # A-21236). All secondary antibodies were diluted at 1:200. All cells except those stained for BrdU were counterstained with DAPI NucBlue Fixed Cell Stain (Molecular Probes, # R37606).

### Confocal Microscopy

Immunolabeled cells were imaged by laser-scanning confocal microscopy (LSM-710, Zeiss, Jena, Germany) configured around an AxioObserver 21 (inverted) stand with a motorized XY stage. Image acquisition was performed using the Zen Black edition (Carl Zeiss, 2011; 64 bit, version 8.1.5.484) software package. 16-bit images were acquired with a 20x/0.8 NA plan apochromat objective lens, with a pixel dwell time of 0.99 μsec and a pixel size of 0.13 μm^2^. Images were acquired using 4x line averaging, with simultaneous scanning of the 405 Diode and 633 HeNe lasers, and a sequential scan for the 488 Argon and 561 DPSS lasers. The 488 laser line was also used to generate a transmitted light DIC image. At least 5 fields of view (424.84 μm^2^) were acquired for each condition (EF strength x time), and each experiment was repeated at least 3 times. Detector windows for each channel were adjusted to assure no cross talk between channels as follows: 405 nm (410–483 nm), 488 nm (492–560 nm), 561 nm (580–629 nm), and 633 nm (637–735 nm).

### Image Analysis

Images were imported into Fiji (an ImageJ distribution built for the Life Sciences; http://fiji.sc/Fiji) [126] for quantifying cellular and nuclear morphology. To assess alignment of cell processes, gray-scale images of Vimentin expression were analyzed using the 2D Fast Fourier Transform (FFT) algorithm and Oval Profile plugin (authored by Bill O’Connell, http://rsb.info.nih.gov/ij/plugins/oval-profile.html) as described previously [127–129]. The 2D FFT produces an image that is the graphical representation of the spatial frequencies of the original images, which is related to directionality. With the Oval Profile Plugin, the radial summation of pixel intensities is used to determine whether these pixels are randomly distributed around the axis (i.e. are unaligned), or show clustering around a particular orientation (i.e. demonstrate alignment). The pixel intensities (in arbitrary greyscale units) are normalized for each image by dividing the value at each angle measure by the minimum radial pixel intensity sum for that image and then subtracting 1). Normalized pixel intensities for each angle measure in the oval profile are averaged across all images acquired from each group, and then those averaged values were normalized again. A graphical representation of orientation in the original image is obtained by plotting the summed pixel intensities between 0° and 180° (the directionality information is axial and does not distinguish between objects pointing in opposite directions; the data were plotted from 0° to 360° because double-plotting the data helped aid in visualizing directionality). It should be noted that the FFT image was first rotated 90° counterclockwise because the results of the FFT yields frequencies orthogonal to those in the original image. In our experiments, this rotation also defines the direction of the electric field application along the 0–180° axis (horizontal).

### Fluorescence Microscopy and Proliferation Assay

Astrocytes were exposed to an electric field of either 0, 4, 40, or 400 mV/mm for either 12, 24, 48, or 72 hours. BrdU was added to the culture media for the last 6 hours of EF exposure. At the end of the experiment, the cell cultures were fixed with 4% paraformaldehyde and then immunolabeled for BrdU. Digital images of BrdU-immunolabeled cells were acquired with a 25x/0.8 NA Plan-Neofluar objective lens using DIC optics and a GFP filter cube (filter set FS 38HE, Zeiss, Jena Germany) using a Zeiss Axiovert 200 inverted microscope (Zeiss, Jena Germany) equipped with a Hamamatsu ORCA ER CCD camera, Colibri LED illumination unit (blue, green, red), and a white light LED. Image acquisition was performed using the Zeiss Axiovision (version 4.8.2 sp1) software package. At least 20 fields of view were randomly acquired for each slide, allowing at least 1,000 cells to be counted for each group. The number of BrdU-positive or negative cells were counted using the Cell Counter plugin for Fiji (authored by Kurt De Vos, http://rsb.info.nih.gov/ij/plugins/cell-counter.html). For each experiment, we compared our hypothesis that EFs induce proliferation at each time point against the null hypothesis that they have no effect on proliferation using a test of homogeneity of proportions; if a significant effect was detected, we then performed individual X^2^ tests between EF strengths to determine which specific groups were different. Results from individual experiments were used to develop a sense of trends of how EFs affect proliferation over time. To evaluate the effects that EF exposure has on proliferation at each time point, we compiled the percentage of BrdU-positive cells from each of the individual experiments and compared these percentages between EF exposures using a Kruskal-Wallis test (*p* < 0.05 at each time point) with nonparametric comparisons between each EF exposure and the 0 mV/mm control at each time point using the Dunn Method for Joint Ranking.

### Statistical Analysis

All data analyses and graphing were performed using R (including packages Circular, Ggplot2, Pastecs, Reshape, and Multcomp) [119–124], with RStudio [125]. Directional data were evaluated using the Rayleigh test, which tests the research hypothesis of non-random directionality against a null hypothesis of random directionality based on the test statistic of the mean resultant vector (*R*). Circular statistics, including the circular mean direction (*μ*), circular standard deviation, and concentration parameter (*κ*), were calculated using the R package Circular [120]. For all experiments, the nominal threshold for significance was set at *p < 0.05, unless otherwise noted. Unless otherwise noted, data are reported as mean ± SEM. All figures were prepared using the ImageJ plugin FigureJ [130].

## Results

### Characterizing the astrocytic population

**Fig 3:**
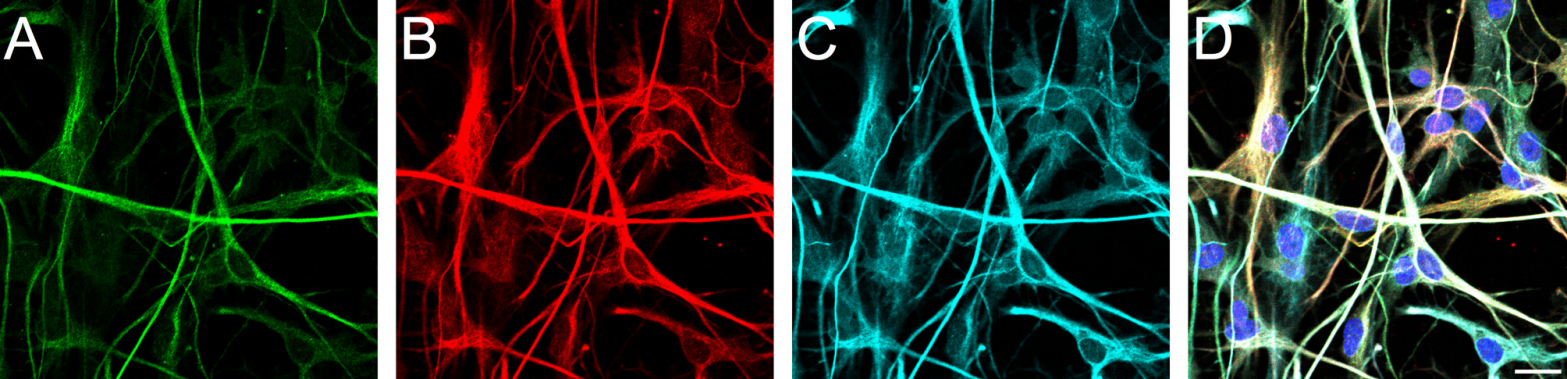
Characterizing the astrocyte population. Representative confocal images used to characterize the astrocyte population based on immunolabeling. (A) GFAP, (B) vimentin, (C) nestin, and (D) an overlay. Scale bar = 20 μm.

The cortical astrocytes (rat primary cultures) used in these experiments were purified populations (>99%) as verified by the provider (ScienCell), with GFAP staining. Immunofluorescence labeling against GFAP, vimentin, and nestin, as well as morphological characteristics as visualized with DIC microscopy, were used to evaluate the purity and maturation of these astrocytes before each experiment (Fig 3). These cultured astrocytes expressed GFAP at varying levels of intensity, while vimentin and nestin were more consistently expressed in all cells. Morphologically, the astrocytic population is heterogeneous, consisting of both lamellipodial and process-bearing cells. Together, this confirmed that >99% of the cells were astrocytes at varying degrees of maturation.

### Elevated electric fields affect the speed of astrocyte migration

To test our overall hypothesis that electric fields are capable of directing the astrocytic response to injury, we explored how EF exposure affects each of the characteristic behaviors that astrocytes display after injury in both mammalian and non-mammalian vertebrates. The first of these behaviors is migration, as astrocytes must move towards the lesion as they are recruited to restore BBB integrity and isolate the lesion environment from the surrounding healthy tissue. To assess the extent to which EFs influence astrocyte migration, time-lapse DIC live cell microscopy was used to record the astrocytic response to direct current electric field exposure over a 12–15 hour period (S1–S4 Videos). The migration of astrocytes following exposure to EF intensities associated with intact (4 mV/mm), injured mammalian (40 mV/mm), and injured non-mammalian vertebrate tissues (400 mV/mm) were compared to an untreated control (0 mV/mm). No evidence of cell death was found during these experiments as a function of either EF exposure or of phototoxicity from repeated exposure to light.

Time-lapse videos show that, in the absence of any EF (S1 Video), astrocytes displayed heterogeneous morphologies (bipolar, stellate and lamellipodial) and displayed non-directional movement. Cells exposed to 4 mV/mm (S2 Video) showed similar morphologies but displayed reduced movement as compared to astrocytes cultured in the absence of any EF. In contrast, time-lapse videos showed that cells exposed to 40 mV/mm (S3 Video) or 400 mV/mm (S4 Video) responded rapidly to the EF exposure, with the entire cell population migrating towards the anode of the EF within the first hour of the EF exposure.

**Fig 4:**
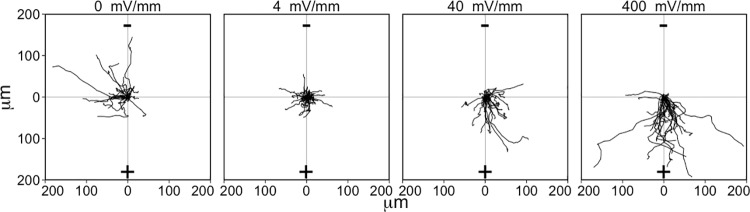
Paths of astrocyte migration over the first 6 hours of EF exposure. These graphs demonstrate the different effect that each EF strength has on directional migration. Each line represents the path of migration of an individual cell, with the starting point normalized to the origin (center) of the graph (0, 0). The cathode (+) is at the top of the graph and the anode (-) is at the bottom of the graph. 30 cells from each EF strength were randomly selected to be included in this plot (including more than 30 cells makes it difficult to discern individual tracks). Cell migration paths were only plotted for cells that remained in the field of view throughout the first 6-hours of the experiment. X- and Y-axis units for the graph are in micrometers.

**Fig 5:**
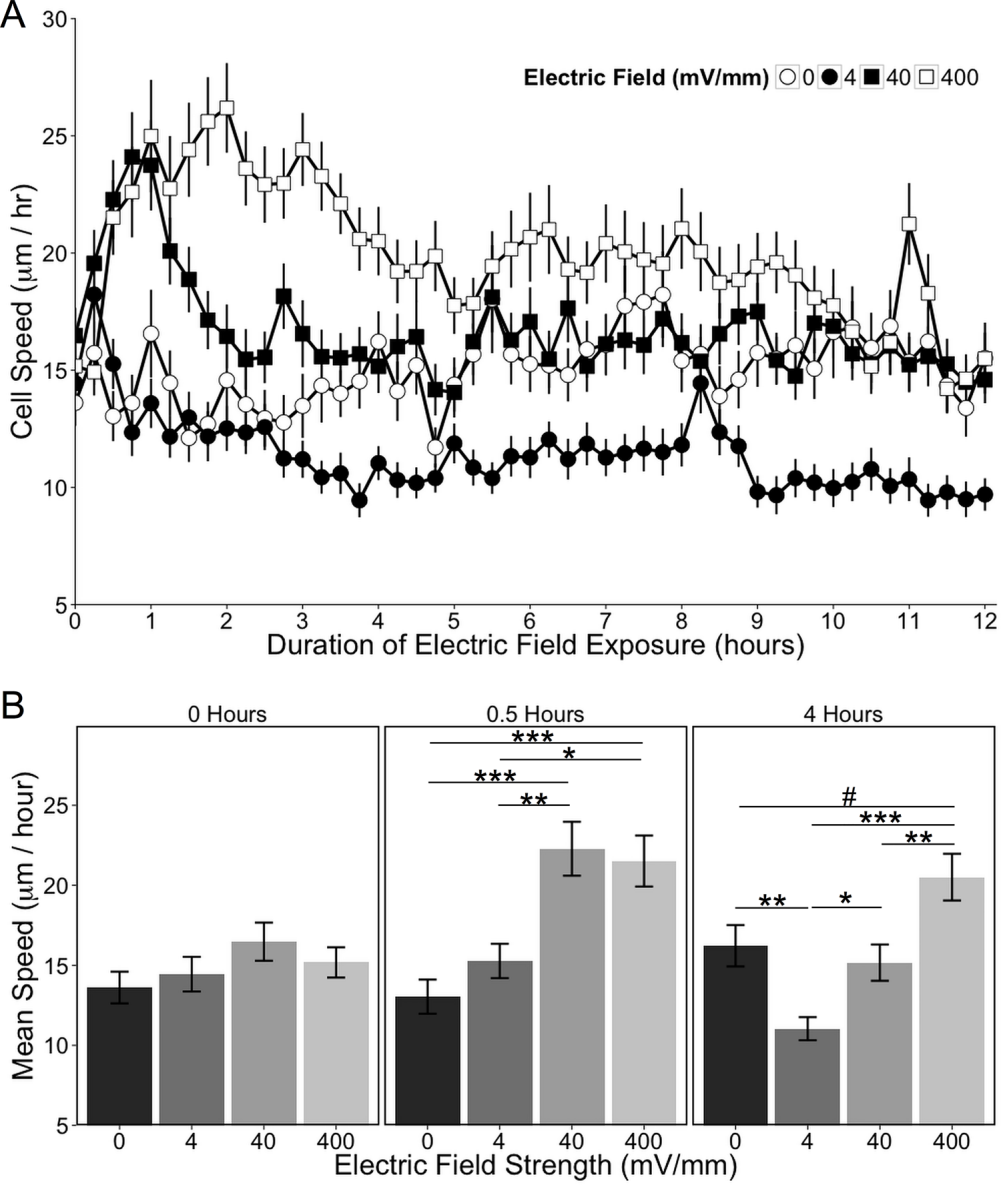
Electric field exposure affects astrocyte migration speed. (A) Astrocyte migration speed is plotted over time, with mean speed measured every 15 minutes for 12 hours. (B) Effects of EF exposure on speed were assessed at each time point and 3 representative time points are shown corresponding to the start of the experiment (0 hours), and 30 minutes and 4 hours after EF onset. Mean speed was compared between EF strengths at each time point with a one-way ANOVA followed by a Tukey-HSD post hoc comparison. All data are expressed as mean ± SEM. #*p* = 0.0509; *p < 0.05; **p < 0.01; ***p < 0.001.

To qualitatively evaluate the EF-induced effect on migration, the paths of migration over the first six hours of EF exposure were plotted for individual astrocytes relative to the orientation of the EF (Fig 4). An analysis of the mean speeds for astrocytes exposed to each of the EF intensities showed that the mean speed of cells in the control group (0 mV/mm) did not change over time, while there were different effects on speed for each of the EF exposures (Fig 5A). Differences in cell speeds were compared among all EF exposure groups at each time point using a 1-factor ANOVA with a Tukey-HSD post-hoc test (Fig 5B graphs this analysis for cells at the start of the experiment, and after 30 minutes and 4 hours of EF exposure). We found that the mean cell speed was equivalent among all groups prior to the EF onset (1-factor ANOVA: *F*_(3, 471)_ = 1.54, *p* = 0.20; mean speed ± SEM: 0 mV/mm: 13.6 ± 0.99 μm/hr; 4 mV/mm: 14.4 ± 1.08 μm/hr; 40 mV/mm: 16.5 ± 1.19 μm/hr; 400 mV/mm: 15.2 ± 0.95 μm/hr; Fig 5B, left panel). However, astrocytes exposed to 40 and 400 mV/mm displayed a rapid increase in migration speed within 30 minutes of EF exposure as compared to astrocytes exposed to 0 or 4 mV/mm (1-factor ANOVA: *F*_(3, 474)_ = 10.5, *p* = 1.1 x 10^−6^; mean speed ± SEM: 0 mV/mm: 13.0 ± 1.07 μm/hr; 4 mV/mm: 15.3 ± 1.07 μm/hr; 40 mV/mm: 22.3 ± 1.68 μm/hr; 400 mV/mm: 21.5 ± 1.59 μm/hr; Fig 5B, center panel). Interestingly, astrocytes exposed to 40 mV/mm sustained this increased speed for only one hour and returned to the same speed as astrocytes exposed to 0 mV/mm 1.75 hours after the EF onset. In contrast, astrocytes exposed to 400 mV/mm sustained an increased speed for over 4 hours, after which time it decreased towards baseline and maintained an elevation that hovered between statistically significant and a non-significant trend. Astrocytes exposed to 4 mV/mm did not show an initial change in migration speed upon EF exposure, but the mean speed decreased relative to cells exposed to 0mV/mm beginning 3.5 hours after the EF onset. EF-induced changes in migrational speed became stable after 4 hours (1-factor ANOVA: *F*_(3, 483)_ = 11.8, *p* = 1.8 x 10^−7^; mean speed ± SEM: 0 mV/mm: 16.2 ± 1.29 μm/hr; 4 mV/mm: 11.0 ± 0.72 μm/hr; 40 mV/mm: 15.2 ± 1.13 μm/hr; 400 mV/mm: 20.5 ± 1.46 μm/hr; Fig 5B, right panel) and persisted for the remainder of the experiment. Thus, EF intensities comparable to those present in intact tissue induced cortical astrocytes to decrease their speed, whereas EF intensities comparable to those present in injured mammalian tissue initiated a rapid increased speed of migration by these cells. This migrational response was more pronounced and sustained in astrocytes exposed to EF intensities associated with regenerating tissues in non-mammalian vertebrates.

As the migrational assays were done sequentially, it was necessary to assess whether the time interval between when the cells were sub-cultured in the EF chamber and when EF exposure began had any effect on the cellular responsiveness to the electric field. Consequently, sister cultures were exposed to 40 mV/mm for 12 hours, beginning either 16 or 48 hours after the cells were sub-cultured into the EF chamber and migration speed assessed. No difference in the mean migration speed between these groups was found (S1 Fig), indicating that the sequence in which groups were exposed to each EF within an experiment does not serve as a confounding variable in these studies.

### Electric fields are a directional cue for astrocyte migration

**Fig 6:**
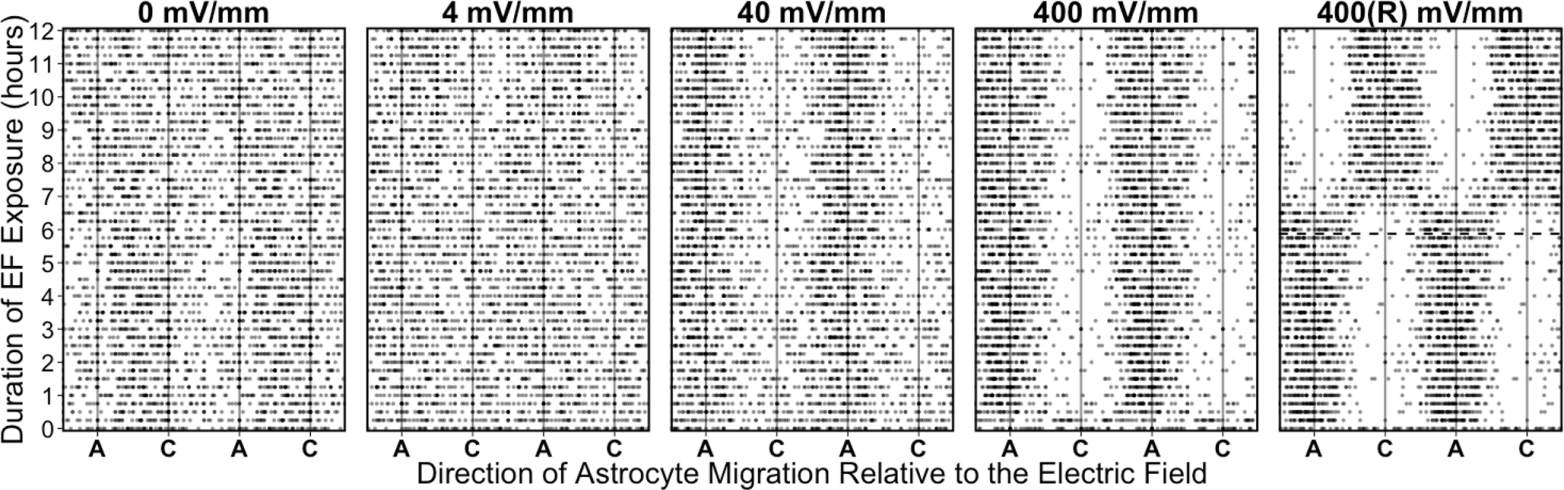
EFs induce directional migration. Astrocytes preferentially migrate towards the anode of an applied EF of 40 or 400 mV/mm. The direction of migration was measured for each cell every 15 minutes over 12 hours relative to the anode (A) and cathode (C) of the applied EF and plotted, with each dot representing the direction of migration of a single cell at each time point. The x-axis is double-plotted for each EF strength to help visualize the directionality. The random direction of cell movement in 0 and 4 mV/mm is visually displayed by the even distribution of data points along the x-axis. Directional migration towards the anode emerges in cells exposed to 40 mV/mm after 1.5 hours. 400 mV/mm induces anodally-directed migration after 30 minutes, which is more concentrated (greater concentration parameter, κ) towards the anode than it is for cells exposed to 40 mV/mm as visually indicated by the stronger clustering of cell directions towards the anode. If the polarity of the 400 mV/mm EF is reversed after 6 hours (panel labeled 400(R) mV/mm, time when current was reversed is indicated with the dashed line), cells reorient to the new EF vector over the following 2 hours.

Having demonstrated that EF exposure alters cell speed in an intensity- and time-dependent manner, we assessed to extent to which EFs also serve as an orientational cue by causing directional migration. Directionality was assessed for each EF intensity at each time point using Rayleigh’s test (which tests the hypothesis of a non-random direction about a circle against the null hypothesis of a random direction), using an overall *p*-value = 0.05 with a Bonferroni correction for the total number of factor levels analyzed (196 comparisons: 4 EF levels at each of 49 time points). No directional migration was detected by astrocytes exposed to EF intensities of either 0 or 4 mV/mm at any time point (Fig 6). In contrast, astrocytes exposed to 400 mV/mm displayed anodally-directed migration within only 30 minutes of exposure (400 mV/mm 30 minutes after EF onset: *R*_106_ = 0.317, *p* = 2.13 x 10^−5^; *μ* = 63.5 ± 12.0°, κ = 0.669 ± 0.148); after 1 hour of exposure, astrocytes continued to migrate towards the anode (*R*_106_ = 0.623, *p* = 9.76 x 10^−19^; *μ* = 71.1 ± 5.55°, κ = 1.6 ± 0.203) with greater precision (i.e. less dispersion) than they had been migrating with after only 30 minutes (equal kappa test: *X* ^2^ = 13.9, *p* = 1.94 x 10^−4^). Astrocytes exposed to 40 mV/mm also displayed anodally-directed migration, but it did not emerge until 1 hour after EF onset (40 mV/mm 1 hour after EF onset: *R*_156_ = 0.232, *p* = 2.21 x 10^−4^; *μ* = 85.2 ± 13.8°, κ = 0.478 ± 0.118) (Fig 6). Once it emerged, directional migration continued throughout the remainder of the recording period. Moreover, astrocytes exposed to 400 mV/mm moved with greater precision towards the anode as compared to astrocytes exposed to 40 mV/mm as demonstrated by a larger concentration parameter (κ) after 1 hour of EF onset (equal kappa test: *X* ^2^ = 27.4, *p* = 1.64 x 10^−7^). This difference in the precision of directional migration persisted at 2 hours (40 mV/mm: κ = 0.477 ± 0.119; 400 mV/mm: κ = 1.75 ± 0.214; equal kappa test: *X* ^2^ = 33.8, *p* = 6.22 x 10^−9^), at 3 hours (40 mV/mm: κ = 0.551 ± 0.117; 400 mV/mm: κ = 1.35 ± 0.183; equal kappa test: *X* ^2^ = 14.7, *p* = 1.28 x 10^−4^), at 4 hours (40 mV/mm: κ = 0.889 ± 0.129; 400 mV/mm: κ = 1.8 ± 0.22; equal kappa test: *X* ^2^ = 14.2, *p* = 1.62 x 10^−4^), and thereafter throughout the duration of the experiment.

As EFs rapidly induced directional migration by cortical astrocytes, we next set out to determine whether the cells remained sensitive to changes in the extracellular EF orientation. This was tested by exposing cells to 400 mV/mm for 6 hours, and then reversing the polarity of the EF exposure for another 6 hours (Fig 6, panel 400(R) mV/mm). We found that, upon reversing the direction of the EF, cells stop moving towards the position that used to be the anode within 15 minutes, and reestablished directional migration towards the new anode position within 2 hours. This 2-hour loss of directionality occurred while the cells were reorienting to the new direction of the imposed EF, during which time half of the population turned clockwise and the other half turned counter-clockwise (as indicated by the phase-shift in the directionality data from hours 6–8; Fig 6, panel 400(R) mV/mm). Together, these results indicate that the cells are capable of detecting the external EF and move towards the anode, with the strength of the EF affecting the directionality and speed of migration.

### Electric fields induce astrocyte proliferation

**Fig 7:**
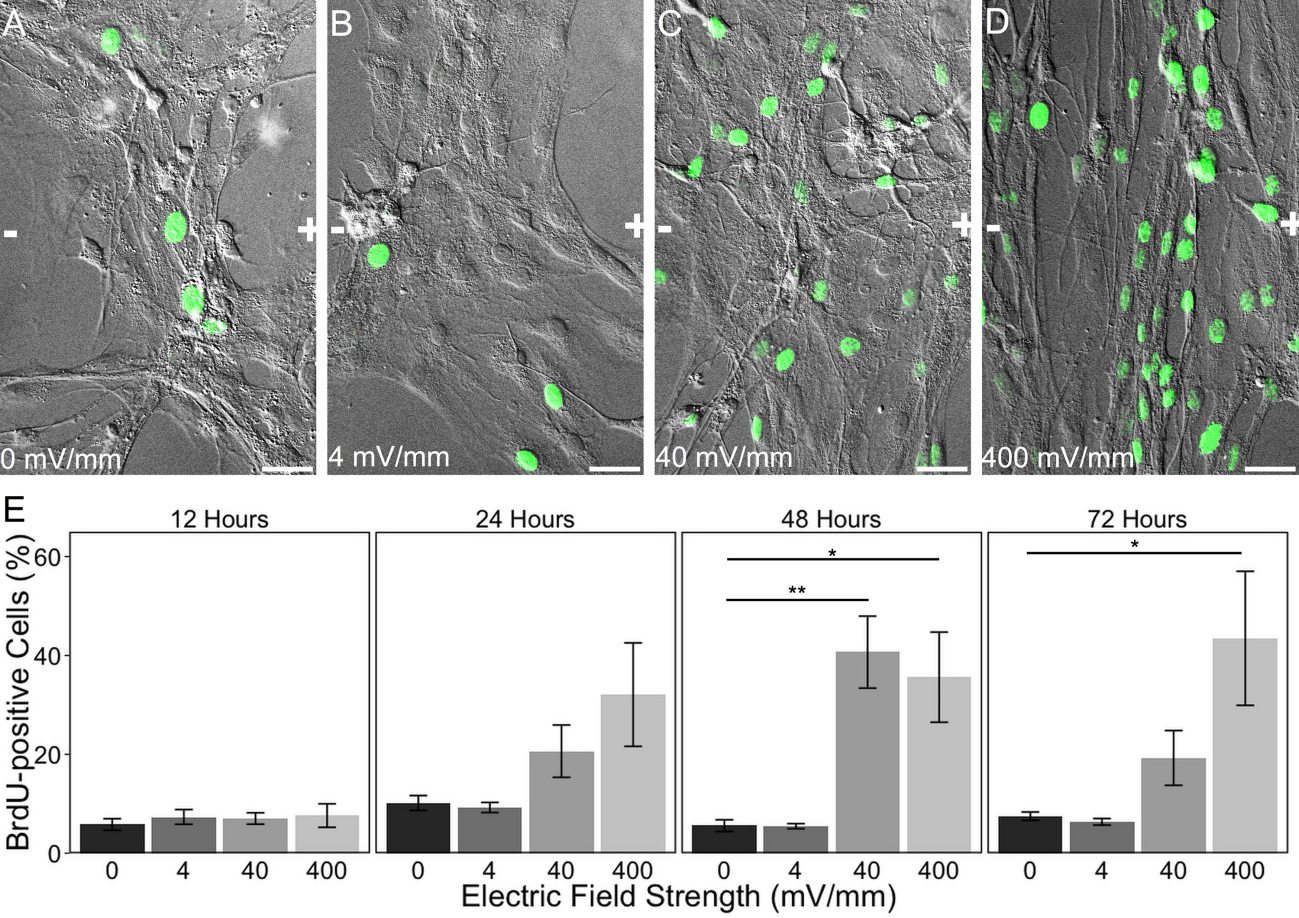
Electric field exposure induces astrocyte proliferation. (A-D) Representative BrdU immunolabeling overlaid on 20x DIC images of cortical astrocytes 48 hours after exposure to 0 (A), 4 (B), 40 (C), or 400 (D) mV/mm. Scale bars: 20 μm. The orientation of the applied EF is indicated in each image, with the anode (+) to the right of the image and the cathode (-) to the left. (E) Quantification of change in proliferation over time as a function of EF strength by comparing the % BrdU-positive cells to the total population of cells present. All data are expressed as mean ± SEM. Kruskal-Wallis test followed by individual nonparametric comparison post-hoc tests using the Dunn Method for Joint Ranking. *p < 0.05, **p < 0.01.

After an injury in the mammalian CNS, astrocytes around the lesion site proliferate with a well-described time course that begins within 24 hours of the injury, peaks after 48 hours, and begins to decline by 72 hours [5,6,8,15]. This newly-proliferating population helps reestablish the damaged blood brain barrier [11,12,131], and serves to replenish some of the cells lost to injury. We tested the hypothesis that EFs associated with injured tissues (40, 400 mV/mm) may actually drive this proliferative response. Specifically, astrocytes were exposed to an EF of either 0, 4, 40, or 400 mV/mm for either 12, 24, 48, or 72 hours to determine whether EFs can induce astrocyte proliferation, and whether this effect mirrors the temporal profile of the proliferative response of astrocytes following injury *in vivo*. BrdU was added to the culture media for the last 6 hours of the EF exposure, and proliferating astrocytes were identified using BrdU immunocytochemistry (Fig 7A–7D). Cells were counted as either BrdU-positive or negative, and the percentage of BrdU-labeled astrocytes was calculated for each factor level (4 EF strengths x 4 time points) within each experiment. The percentage of BrdU-labeled astrocytes was compared among the EF-exposure groups within each time point using a Kruskal-Wallis test, with a Wilcoxon test for post-hoc comparisons between each EF exposure group and the 0 mV/mm control (Fig 7E).

At all 4 time points, 5–10% of cells exposed to 0 mV/mm were BrdU-positive, and there was no statistically significant difference in BrdU labeling of these cells over time (Kruskal-Wallis: X^2^_3_ = 6.68, *p* = 0.828). For all EF strengths, there was no change in the percentage of astrocytes labeled with BrdU after 12 hours’ exposure (Kruskal-Wallis: X^2^_3_ = 0.7400, *p* = 0.8638); there was still no change after 24 hours (Kruskal-Wallis: X^2^_3_ = 4.4643, *p* = 0.2155), although a trend towards increased proliferation began to emerge for astrocytes exposed to 40 and 400 mV/mm that was likely non-significant because of the conservative nature of the non-parametric statistical test used. After 48 hours of astrocyte exposure to either 40 or 400 mV/mm EF, there was a statistically significant effect on proliferation (Kruskal-Wallis: X^2^_3_ = 13.5526, *p* = 0.0036), with a significant increase in astrocytes exposed to 40 mV/mm (Wilcoxon: *p* = 0.0088) and 400 mV/mm (Wilcoxon: *p* = 0.0481) relative to 0 mV/mm; in contrast, 4 mV/mm had no effect on proliferation (*p* = 1.0000). After 72 hours, there was still an observed increase in proliferation (Kruskal-Wallis: X^2^_3_ = 13.0060, *p* = 0.0046), but only cells exposed to 400 mV/mm remain elevated (Wilcoxon: *p* = 0.0386) relative to 0 mV/mm (Wilcoxon test: 4 mV/mm: *p* = 1.0000; 40 mV/mm: *p* = 0.1452). Thus, EFs are capable of stimulating proliferation by astrocytes in an intensity- and time-dependent manner, and the time-course over which proliferation increases mirrors that observed for astrocytes at sites of injury to the CNS. EFs associated with injured mammalian tissues (i.e. 40 mV/mm) induce astrocyte proliferation *in vitro* that peaks at 48 hours and declines by 72 hours, consistent with the peak in astrocyte proliferation following cortical injury that has been reported in mammals *in vivo*. Moreover, EFs associated with regenerating non-mammalian vertebrate tissues (i.e. 400 mV/mm) induce astrocyte proliferation *in vitro* at 48 hours that is sustained through 72 hours, consistent with the sustained increase in astrocyte proliferation throughout regeneration in non-mammalian vertebrates that has been reported *in vivo* [7,22]. Thus, EFs associated with injured mammalian tissues may contribute to the proliferative astrocytic response to injury, while EFs associated with regenerating non-mammalian vertebrates can modify astrocyte proliferative activity towards that associated with successfully regenerating animals.

**Fig 8:**
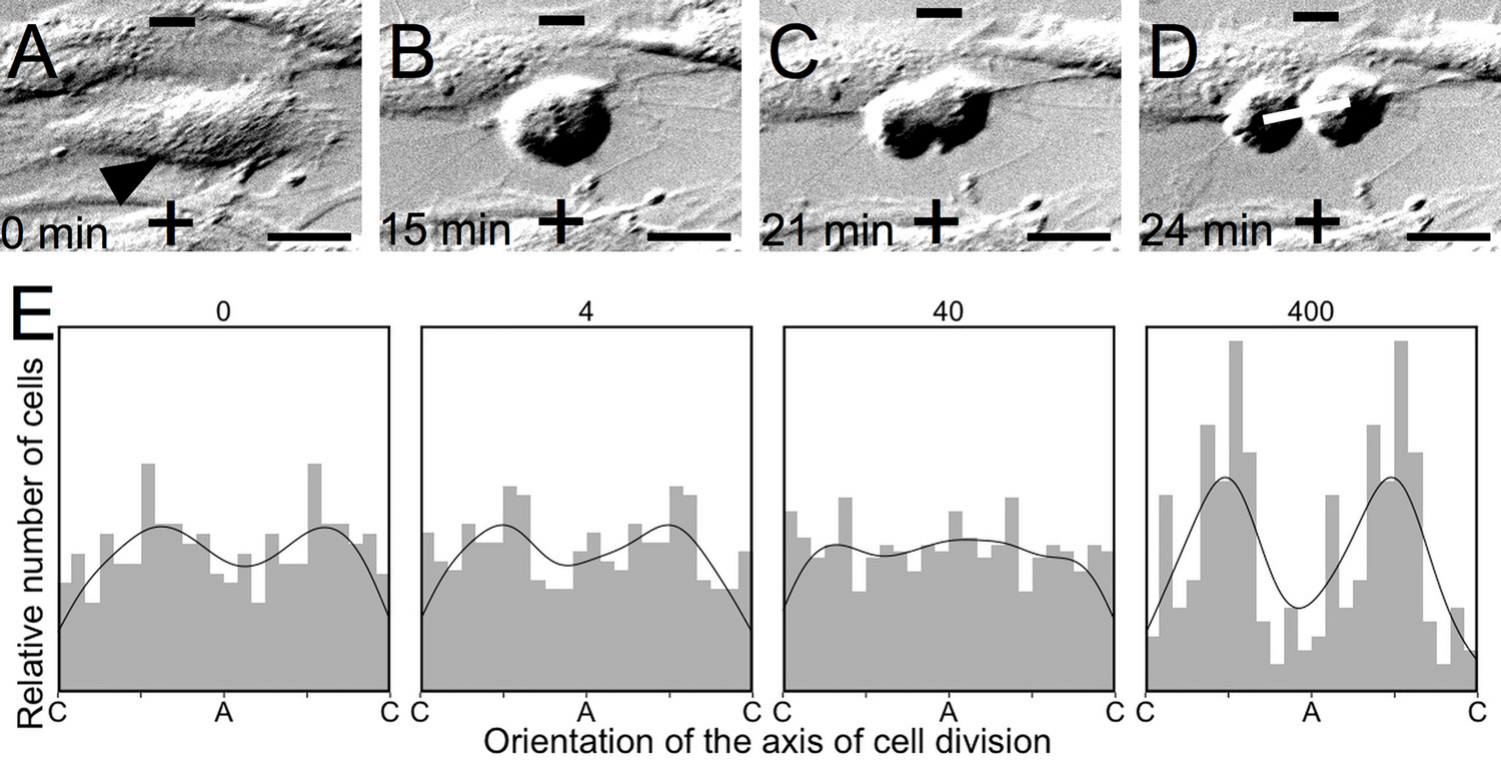
Electric field exposure aligns the axis of cell division. (A-D) Mitotic cells (arrow, A) were identified in time-lapse DIC microscopy videos. The axis of division was defined for each cell by drawing a line through the center of each of the daughter nuclei in the first frame where they become distinctly visible (line, D) and measuring the angle of this axis relative to the applied EF (cathode (-) at the top of each image, anode (+) at the bottom of each image). The time (in minutes) of each image is provided to illustrate the duration of mitosis. Scale bar = 20 μm. (E) Frequency histograms representing the relative number of nuclei counted at each orientation relative to the anode (A) and cathode (C) (histogram bin width of 15°), with a density curve super-imposed on each graph. Orientation of the mitotic axis is double-plotted along the x-axis relative to the anode (A) and cathode (C) to aid in observing the alignment of these data.

One additional observation that was apparent in the time-lapse videos of astrocyte cultures exposed to 400 mV/mm EF is that the axis of division was related to the orientation of the EF vector. As the orientation of division is known to influence cellular activity and the subsequent fate of daughter cells in the developing neural tube during embryogenesis [132], we set out to determine the extent to which EF exposure influences the axis of division in mammalian astrocytes. Using the DIC time-lapse videos, mitotic cells were identified and the angle of the axis of division was measured relative to the EF vector by drawing a line between the two daughter nuclei in the first frame where they became distinctly visible (Fig 8A–8D). The distributions of these axes relative to the EF vector are plotted for each EF strength (Fig 8E). Each EF strength was evaluated for alignment with Rayleigh’s test, and we found that only 400 mV/mm induced alignment (n = 124, R = 0.3740, *p* = 2.93 x 10^−8^), with a mean direction μ ± SEM = 82.1 ± 3.53°, κ = 2.71, and SD = 39.7° (the EF axis runs from 0° to 180°, with 90° being perpendicular to the EF vector). There was no significant alignment for cells exposed to 0 mV/mm (n = 176, R = 0.1127, *p* = 0.1071), 4 mV/mm (n = 186, R = 0.1071, *p* = 0.1183), or 40 mV/mm (n = 260, R = 0.0437, *p* = 0.6092).

### Electric fields alter astrocyte morphology

**Fig 9:**
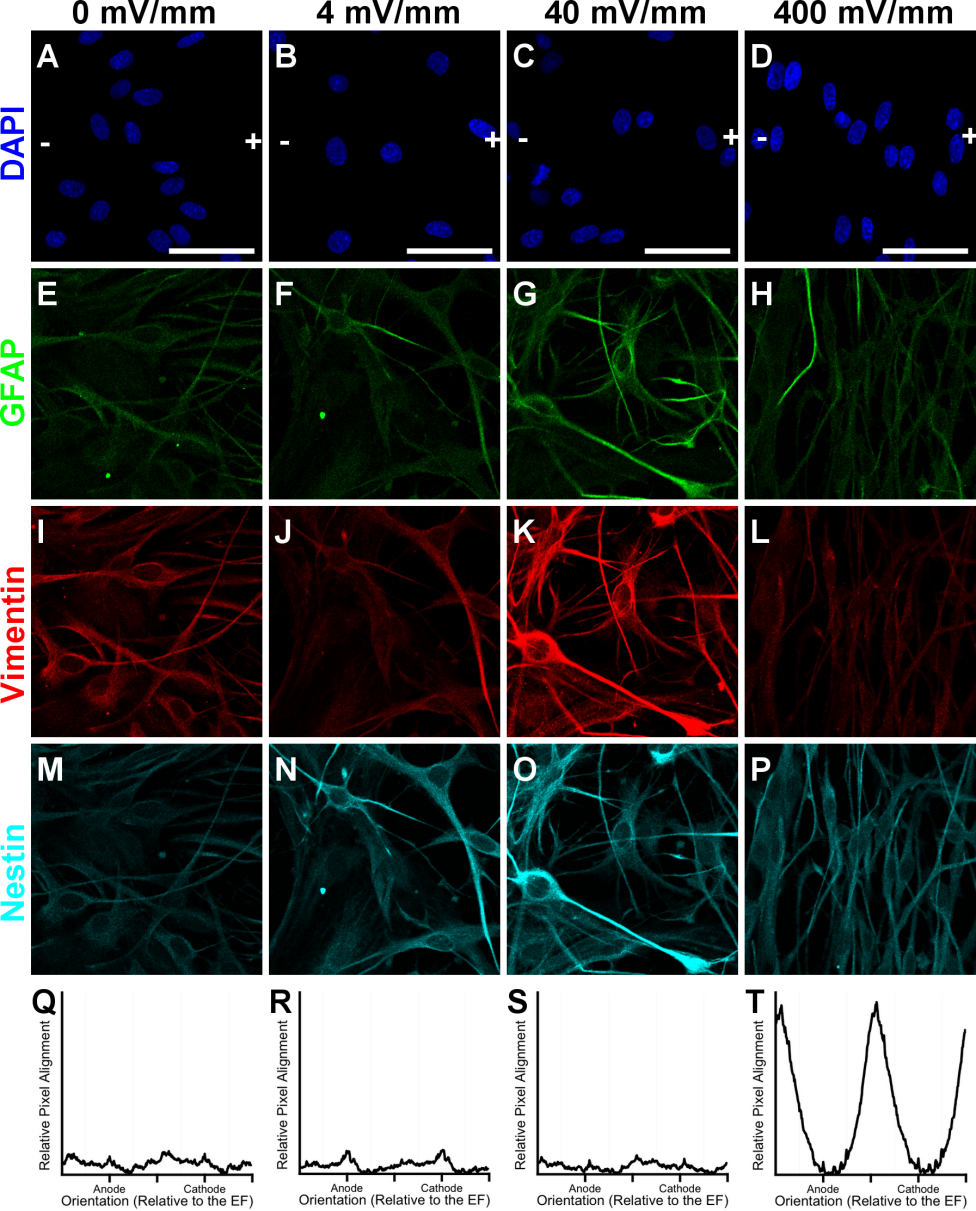
EF effects on intermediate filament expression and morphology. Representative confocal images of astrocytes after 72 hours’ exposure to 0, 4, 40, or 400 mV/mm. (A-D) DAPI-labeled nuclei, with an overlay indicating the orientation of the EF vector (+) and (-), corresponding to the anode and cathode, respectively. Immunolabeling for GFAP (E-H), vimentin (I-L), and nestin (M-P). (Q-T) FFT analysis of normalized pixel intensity from vimentin-labeled images (averaged over 6–8 images) plotted as a function of direction relative to the anode and cathode. The high peaks for astrocytes exposed to 400 mV/mm demonstrate that astrocytes preferentially align their processes perpendicularly to the EF vector, while the absence of peaks for astrocytes exposed to 0, 4, or 40 mV/mm demonstrates the random alignment of their processes. All FFT graphs are plotted with the same scale on the vertical axis; the horizontal axis indicates directionality relative to the anode (+) and cathode (-). Scale bar: 50 μm.

Having shown that EFs can control behaviors that are necessary for the initial astrocytic recruitment to the injury response, we tested the hypothesis that EFs can also regulate the hypertrophic and morphologic changes characteristic of the astrocytic response to injury in non-regenerating and regenerating animals, respectively. Following an injury, astrocytes in non-regenerating animals characteristically up-regulate the expression of the intermediate filament GFAP, relative to the intermediate filaments vimentin and nestin, while astrocytes in regenerating animals do not undergo this hypertrophic change [4,5,7,10,13,16]. As this response is delayed, we exposed astrocytes to 0, 4, 40, or 400 mV/mm for 72 hours, and then used immunofluorescence labeling for GFAP, vimenin, and nestin to determine whether EFs affect hypertrophy (Fig 9A–9P). We found that astrocytes exposed to 0 mV/mm, which is the unexposed control, expressed low levels of GFAP, with greater intensity of both vimentin and nestin. Astrocytes exposed to 4 mV/mm, which is an EF-intensity associated with uninjured tissues, had an expression pattern of intermediate filaments very similar to that for the unexposed controls. However, astrocytes exposed to 40 mV/mm displayed elevated levels of both GFAP and vimentin, suggesting that EFs associated with injured mammalian tissues caused robust hypertrophy. Interestingly, we found that GFAP and vimentin expression in astrocytes exposed to 400 mV/mm was unchanged compared to that observed for astrocytes exposed to 0 or 4 mV/mm. Thus, EFs associated with injured mammalian tissues are sufficient to induce a hypertrophic response characteristic of reactive gliosis, while 400 mV/mm induces no such change.

Having found that EF strengths associated with non-regenerating tissues induce cytoskeletal hypertrophy of astrocytes, we next set out to determine whether EFs associated with regeneration induce morphological changes in astrocytes consistent with their regenerative phenotype *in vivo*. Our group and others have shown that astrocytes align their processes perpendicularly to a 500 mV/mm applied EF within 24 hours, and we have previously shown that these EF-aligned astrocytes enhance the extent of neurite outgrowth compared to unaligned 0 mV/mm controls [112,115,116]. Our time-lapse videos from the migration studies indicate that EF exposure to 400 mV/mm induces astrocytes to transform into a bipolar morphology and to align their processes within the first 12 hours of EF exposure. In the current study, we exposed astrocytes to 0, 4, 40, or 400 mV/mm for either 12 or 72 hours and performed FFT analysis for alignment on vimentin immunolabeled images to determine how quickly alignment occurs, whether this alignment is consistent over time, and whether physiologic EFs found at the injury site in mammals affects process alignment (Fig 9Q–9T). Astrocytes exposed to 0, 4, or 40 mV/mm EF showed no change in their alignment at either 12 or 72 hours of exposure. However, astrocytes exposed to 400 mV/mm showed a robust alignment of their processes perpendicular to the electric field orientation within 12 hours (S2 Fig) and this alignment persisted through the 72 hour EF exposure (Fig 9T). These results demonstrate that only EF intensities associated with regenerating non-mammalian vertebrates induce dramatic changes in astrocyte morphology that mirror those demonstrated by astrocytes within the injury site, which facilitates regeneration in these animals following trauma.

## Discussion

Physiologic electric fields have an intensity-dependent effect on wound repair [28,50,77,79,83,99,101,102,110]. A 50- to 100-fold EF increase in non-mammalian vertebrates induced by injury to the skin [54–56], bone [57], cornea [58,59], lens [60–62], spinal cord [63], tail [64,65], and limb [66–71] is necessary and sufficient for regeneration [66,72–83]. In contrast, EFs only increase 10-fold upon injury in mammalian skin [29,52,53,84,85], respiratory epithelium [86], cornea [30,40,59], lens [87,88], bone [57,89,90], and finger amputation [91] where wounds health through scar formation, but increasing these EFs towards levels found in regenerating animals induces a more robust regenerative response [40,79,80,92–94]. The mammalian CNS also produces EFs [51], which increase only 10-fold upon injury [95,97–99,101], and previous research has suggested that EFs associated with regenerating tissues in non-mammalian vertebrates may regulate CNS regeneration by influencing axon outgrowth [47,49,74,103–114], and neural progenitor cell migration and differentiation [33,117,133–136]. Although the astrocytic response to injury determines whether sprouting axons are capable of regenerating past a lesion site, the ability of EFs to regulate this astrocytic behavior has not been explored. We tested the hypotheses that physiologic EFs produced by the injured mammalian CNS are sufficient to induce behaviors associated with the astrocytic response to injury, and that increasing these EFs to levels found in regenerating non-mammalian vertebrates will modify the behavioral response towards one associated with a regenerative phenotype *in vivo*. We evaluated how mammalian astrocytes respond to EF exposures within the ranges previously recorded in intact tissues (4 mV/mm) [51,52], injured mammalian tissues (40 mV/mm) [40,59], and regenerating non-mammalian vertebrate tissues (400 mV/mm) [54,63,67,73,137]. Specifically, we hypothesized that both of the injury-associated EFs would induce behaviors characteristic of the astrocytic response through which they restore the BBB integrity, but that only astrocytes exposed to 400 mV/mm would demonstrate a more robust, long-lasting behavioral response that is associated with successful regeneration.

Our results demonstrate that EFs are capable of inducing multiple behaviors associated with the astrocytic response to injury. Moreover, the EF intensity determines whether the induced behaviors reflect those associated with glial scar formation (i.e. cytoskeletal hypertrophy [1,5,13–15]) or regeneration (i.e. process alignment [4,25]). We found that the only behavioral effect induced by the lowest EF strength was a decrease in migrational speed; 4 mV/mm represents the EF within the intact adult CNS [51] where astrocytes are not migratory and are thought to remain in distinctly defined domains [16], so the decreased speed that this EF exposure induced in astrocytes suggests that these low level EFs may sustain a quiescent non-migratory state among astrocytes *in vivo*. In contrast, both 40 and 400 mV/mm induced anodally-directed migration within an hour of EF onset, but 400 mV/mm induced migration with significantly greater speed and a more precise directionality than that induced by 40 mV/mm. As previous literature suggests that the injury site in the mammalian cortex becomes the anode of the injury-induced EF [51,63,97,98], endogenous EFs may thus recruit astrocytes to respond to the injury by inducing their migration towards the lesion *in vivo*. We also found that EFs have an intensity-dependent effect on proliferation: both 40 mV/mm and 400 mV/mm induce a robust proliferative response that begins to emerge after 24 hours and peaks after 48 hours of exposure; at 72 hours, proliferation decreases towards baseline for astrocytes exposed to 40 mV/mm, but proliferation was sustained at an elevated rate for those exposed to 400 mV/mm. This proliferative response closely follows the temporal profile of astrocyte proliferation following an injury *in vivo*, which peaks two days after injury in both mammalian and non-mammalian vertebrates but is sustained at subsequent time points only in non-mammalian vertebrates [4–15]. Moreover, only EFs associated with regeneration induced morphological changes in astrocytes that mirror those that facilitate regeneration in non-mammalian vertebrates [4,7,25]; in contrast, astrocytes exposed to EFs associated with injury in mammals maintained a heterogeneous morphology and instead demonstrated cytoskeletal hypertrophy that is associated with glial scar formation and the failure of regeneration *in vivo* [1,5,13,15]. The physiologic relevance of EFs as the stimulus for the astrocytic response to injury is supported by our observations that these EF-induced behaviors emerged along the same time line as they emerge following an injury *in vivo* [4,6–8,10,14,15]. Thus, injury-induced EFs may be capable of, and responsible for, driving the astrocytic response to injury and determining the regenerative potential of the vertebrate CNS. Consequently, manipulating the EF intensity in the CNS may represent a therapeutic strategy to promote CNS regeneration.

Based on the criteria for causality as defined by Hill in 1965 [138], our hypothesized causal relationship between EFs and each of the astrocytic behaviors associated with the response to injury is independent of identifying the physiologic mechanisms underlying these effects. The scope of these experiments was thus intentionally limited to EF-induced astrocytic behaviors–migration, proliferation, hypertrophy, and process alignment–because identifying the underlying mechanisms is ancillary to first establishing that EFs regulate each of these behaviors. Having now established that EFs induce each of these behaviors, the plausibility of our hypothesis that EFs regulate the regenerative potential in the mammalian CNS through their influence over these behaviors will be supported by better understanding how astrocytes may transduce EFs.

The EF vector conveys information about both orientation and intensity, each of which can be transduced independently each of which cells may transduce independently through nonspecific electrostatic interactions with their membrane proteins [31,32,39,50,77,102,139]. External EFs induce a proportional change in the activity of voltage-sensitive membrane proteins by affecting the cell’s resting membrane potential (V_m_) [39,96,139,140]; although EFs may only have a small effect on V_m_, small changes in V_m_ can have large changes in ion channel conductance and intracellular ion concentration because many of the ion channels that determine V_m_ are themselves affected by both pH and V_m_ and thus the relationship between V_m_ and the activity of voltage-sensitive membrane proteins is non-linear [39,141]. EFs also induce electroosmosis, the process through which electrostatic forces redistribute charged membrane proteins to either the anodal or cathodal side of the cell [42,48,135,142–145]; each protein’s net charge determines the side of the cell to which it redistributes and thus allows the cellular transduction of EF orientation, while the degree of membrane protein clustering is proportional to the intensity of the external EF [144,145].

Although the particular physiologic pathways through which astrocytes transduce EFs have not been explored, myriad pathways have been implicated in EF transduction in a variety of other cell types and the general trend is that EFs affect cellular behaviors through the same physiologic mechanisms through which these cells otherwise respond to chemical stimuli [27,28,32,135,146]. Directional migration such as we observed in astrocytes exposed to 40 and 400 mV/mm requires the directional extension of cellular processes; localized activation of Ca^2+^ second messenger cascades drives polarization of the Golgi apparatus and microtubule organizing center (MTOC) within the cytosol, facilitating assembly of actin and microtubules beneath the leading edge of the cell and the breakdown of these cytoskeletal elements at the trailing edge of the cell [36,147]. In hippocampal neurons, EFs induce MTOC and Golgi polarization, and thus directional migration, through the antagonistic second messengers phosphoinositide-3-kinase (PI3K) and phosphatase and tensin homologue (PTEN), which become activated at the leading and trailing edges of the cell, respectively [42,135,148]. In astrocytes, EF-induced migration may be mediated by membrane receptors for integrins [149] as well as the transient receptor potential vanilloid-1 [150], as each of these proteins mediates chemotaxis through Ca^2+^-induced protein kinase C signaling at the leading edge of the cell [151–153]. EFs may also influence astrocyte proliferation by affecting V_m_, which exerts a causal role in cell cycle regulation: hyperpolarization induces a reversible mitotic block, while depolarization induces DNA synthesis and mitosis in mature neurons [39,141,154–158]. In mammalian astrocytes, the voltage-gated sodium channel Na_V_1.5 is necessary for astrocyte migration and proliferation after injury: Na^+^ conductance increases, which causes intracellular Ca^2+^ concentration to rise through a Na^+^/Ca^2+^ exchanger and subsequently activates downstream second messenger cascades [159]. In Xenopus tadpoles, another voltage gated sodium channel, Na_V_1.2, is necessary to induce tail regeneration after amputation: increased intracellular Na^+^ activates the salt-inducible kinase (SIK), which subsequently induces multiple pathways associated with regeneration, including Notch; regeneration is lost during a refractory period when Na_V_1.2 expression concurrently declines, but transfecting human Na_V_1.5 rescues regeneration in these animals [160]. The transcriptome of mammalian astrocytes demonstrates that they also express SIK [161], so the same pathway by which EFs regulate regeneration in Xenopus is also conserved in astrocytes.

Our observation that astrocytes exposed EFs in the range associated with regenerating tissues (400 mV/mm) induced cells to divide with an orientation perpendicular to that of the applied EF further supports our hypothesis that EFs may regulate CNS regeneration. The absence of regeneration in the mammalian CNS has been attributed in part to the absence of astrocytic neurogenesis [31,32,162,163]; however, mammalian astrocytes retain a latent capacity for neurogenesis that is regulated by Notch signaling, and astrocytes can function as neural progenitor cells (NPCs) in the hippocampus of the adult mammalian CNS [164–170]. Immature astrocytes also function as NPCs in the embryonic neural tube, and the orientation of their mitotic axis relative to the ventricular surface is important for determining the maturational fate of their daughter cells [171]. EFs over 400 mV/mm affect mammalian hippocampal NPC differentiation [172] and EFs as large as 1800 mV/mm have been measured in the embryonic neural tube [173], so these endogenous EFs may contribute to progenitor differentiation during embryogenesis in part by directing the orientation of the mitotic axis. EFs have been shown to regulate Notch signaling through SIK [160], which is expressed in mammalian astrocytes [161], so EFs may regulate CNS regeneration in part through affecting this latent neurogenic program in astrocytes; therefore, the absence of robust regeneration in the mammalian CNS may be a result of the lower EFs induced by injury being insufficient to reach a threshold of Na_V_1.5 activation that is necessary to regulate Notch-mediated neurogenesis *in vivo*. Thus, the fact that only EFs associated with regeneration induce alignment of the mitotic axis in dividing astrocytes similar to that associated with progenitor cells and neurogenesis supports the hypothesis that these elevated EFs can stimulate a regenerative phenotype in mammalian astrocytes.

We have shown that electric fields within the physiologic ranges reported in injured mammalian tissues and in regenerating non-mammalian vertebrate tissues are able to elicit multiple behaviors in astrocytes that are necessary for their normal response to injury. Furthermore, EFs induce these behavioral changes along the same timeline over which astrocytes express these behaviors following injury *in vivo*. EFs are an ideal physiologic stimulus to drive tissue repair because they are induced immediately upon injury and remain elevated throughout wound healing [50,65]. The physiologic relevance of EFs to the injury response is further supported by our observations that the difference in responses induced by 40 mV/mm and 400 mV/mm closely correspond to the differences between astrocytic behaviors in the injured mammalian CNS and those in the regenerating non-mammalian vertebrate CNS. It has been previously established that elevated EFs promote a regenerative phenotype in neurons by stimulating axon sprouting, and our results expand on this picture by demonstrating that the EF intensity may also determine whether astrocytes inhibit or facilitate axon regeneration past the lesion site. As specific astrocytic behaviors are induced by specific EF strengths, and as behaviors associated with regeneration are induced only at EF strengths greater than those reported in injured mammalian tissues, regeneration in the mammalian CNS may be improved by therapeutically supplementing the physiological EFs produced at the injury site. Together, this suggests that therapeutically-applied EFs are a strong therapeutic candidate to promote regeneration in the mammalian CNS by inducing an astrocytic response more favorable to regeneration.

## Supporting Information

S1 Video Time-lapse video of cortical astrocytes exposed to 0 mV/mm for 12 hours. The time stamp in the top left corner indicates the hours and minutes since the onset of the electric field. The 30 minutes of time-lapse video prior to the onset of the electric field are indicated by negative time stamp (note, astrocytes exposed to 0 mV/mm were recorded for 12.5 hours; the first 30 minutes of this are indicated with a negative time stamp for consistency). The electric field was turned on at 00:00. Each image was taken 3 minutes apart. The anode (+) and cathode (-) of the applied EF are indicated at the bottom and top of the center of the image, respectively. Scale bar (bottom right): 20 μm.
(AVI)

S2 Video Time-lapse video of cortical astrocytes exposed to 4 mV/mm for 12 hours. The time stamp in the top left corner indicates the hours and minutes since the onset of the electric field. The 30 minutes of time-lapse video prior to the onset of the electric field are indicated by negative time stamp (note, astrocytes exposed to 0 mV/mm were recorded for 12.5 hours; the first 30 minutes of this are indicated with a negative time stamp for consistency). The electric field was turned on at 00:00. Each image was taken 3 minutes apart. The anode (+) and cathode (-) of the applied EF are indicated at the bottom and top of the center of the image, respectively. Scale bar (bottom right): 20 μm.
(AVI)

S3 Video Time-lapse video of cortical astrocytes exposed to 40 mV/mm for 12 hours. The time stamp in the top left corner indicates the hours and minutes since the onset of the electric field. The 30 minutes of time-lapse video prior to the onset of the electric field are indicated by negative time stamp (note, astrocytes exposed to 0 mV/mm were recorded for 12.5 hours; the first 30 minutes of this are indicated with a negative time stamp for consistency). The electric field was turned on at 00:00. Each image was taken 3 minutes apart. The anode (+) and cathode (-) of the applied EF are indicated at the bottom and top of the center of the image, respectively. Scale bar (bottom right): 20 μm.
(AVI)

S4 Video Time-lapse video of cortical astrocytes exposed to 400 mV/mm for 12 hours. The time stamp in the top left corner indicates the hours and minutes since the onset of the electric field. The 30 minutes of time-lapse video prior to the onset of the electric field are indicated by negative time stamp (note, astrocytes exposed to 0 mV/mm were recorded for 12.5 hours; the first 30 minutes of this are indicated with a negative time stamp for consistency). The electric field was turned on at 00:00. Each image was taken 3 minutes apart. The anode (+) and cathode (-) of the applied EF are indicated at the bottom and top of the center of the image, respectively. Scale bar (bottom right): 20 μm.
(AVI)

S1 Fig Analysis of sequence effects on astrocyte migration speed. Sister cultures of astrocytes were exposed to a 40 mV/mm EF for 12 hours, with the EF exposure beginning either 16 (labeled 40 mV/mm (1)) or 48 (labeled 40 mV/mm (2)) hours after the cells were sub-cultured. No difference in the mean migration speed between these groups was found, indicating that the sequence in which astrocytes are exposed to each EF within an experiment does not serve as a confounding variable in these studies. Data are plotted as mean ± SEM.
(TIF)

S2 Fig FFT analysis of process alignment after 12 hours of EF exposure. FFT analysis of normalized pixel intensity from vimentin immunolabeled images (averaged over 6–8 images) plotted as a function of direction relative to the anode and cathode. The high peaks for astrocytes exposed to 400 mV/mm demonstrate that astrocytes preferentially align their processes perpendicularly to the EF vector, while the absence of peaks for astrocytes exposed to 0, 4, or 40 mV/mm demonstrates that their processes are not aligned. All FFT graphs are plotted with the same scale in arbitrary units on the vertical axis; the horizontal axis indicates directionality relative to the anode and cathode.
(TIF)

## References

[1] Silver J, Miller JH. Regeneration beyond the glial scar. Nat Rev Neurosci. 2004;5: 146–156. 14735117 10.1038/nrn1326

[2] Tanaka EM, Ferretti P. Considering the evolution of regeneration in the central nervous system. Nat Rev Neurosci. 2009;10: 713–723. 10.1038/nrn2707 19763104

[3] Auguste KI, Jin S, Uchida K, Yan D, Manley GT, Papadopoulos MC, et al Greatly impaired migration of implanted aquaporin-4-deficient astroglial cells in mouse brain toward a site of injury. FASEB J. 2007;21: 108–116. 17135365 10.1096/fj.06-6848com

[4] Goldshmit Y, Sztal TE, Jusuf PR, Hall TE, Nguyen-Chi M, Currie PD. Fgf-dependent glial cell bridges facilitate spinal cord regeneration in zebrafish. J Neurosci. 2012;32: 7477–7492. 10.1523/JNEUROSCI.0758-12.2012 22649227 PMC6703582

[5] Amat JA, Ishiguro H, Nakamura K, Norton WT. Phenotypic diversity and kinetics of proliferating microglia and astrocytes following cortical stab wounds. Glia. 1996;16: 368–382. 8721677 10.1002/(SICI)1098-1136(199604)16:4<368::AID-GLIA9>3.0.CO;2-W

[6] Barreto GE, Sun X, Xu L, Giffard RG. Astrocyte proliferation following stroke in the mouse depends on distance from the infarct. PLoS One. 2011;6: e27881 10.1371/journal.pone.0027881 22132159 PMC3221692

[7] Dawley EM, O Samson S, Woodard KT, Matthias KA. Spinal cord regeneration in a tail autotomizing urodele. J Morphol. 2012;273: 211–225. 10.1002/jmor.11019 21956379

[8] King LA, Mitrophanous KA, Clark LA, Kim VN, Rohll JB, Kingsman AJ, et al Growth factor enhanced retroviral gene transfer to the adult central nervous system. Gene Ther. 2000;7: 1103–1111. 10918476 10.1038/sj.gt.3301198

[9] Kornyei Z, Czirok A, Vicsek T, Madarasz E. Proliferative and migratory responses of astrocytes to in vitro injury. J Neurosci Res. 2000;61: 421–429. 10931528 10.1002/1097-4547(20000815)61:4<421::AID-JNR8>3.0.CO;2-4

[10] Suzuki T, Sakata H, Kato C, Connor JA, Morita M. Astrocyte activation and wound healing in intact-skull mouse after focal brain injury. Eur J Neurosci. 2012;36: 3653–3664. 10.1111/j.1460-9568.2012.08280.x 23013365 PMC5394426

[11] Faulkner J, Herrmann J, Woo M, Tansey K, Doan N, Sofroniew M. Reactive astrocytes protect tissue and preserve function after spinal cord injury. 2004;24: 2143–2155. 14999065 10.1523/JNEUROSCI.3547-03.2004 PMC6730429

[12] Myer DJ, Gurkoff GG, Lee SM, Hovda DA, Sofroniew MV. Essential protective roles of reactive astrocytes in traumatic brain injury. Brain. 2006;129: 2761–2772. 16825202 10.1093/brain/awl165

[13] Okada S, Nakamura M, Katoh H, Miyao T, Shimazaki T, Ishii K, et al Conditional ablation of Stat3 or Socs3 discloses a dual role for reactive astrocytes after spinal cord injury. Nat Med. 2006;12: 829–834. 16783372 10.1038/nm1425

[14] Sofroniew MV. Molecular dissection of reactive astrogliosis and glial scar formation. Trends Neurosci. 2009;32: 638–647. 10.1016/j.tins.2009.08.002 19782411 PMC2787735

[15] Wanner IB, Anderson MA, Song B, Levine J, Fernandez A, Gray-Thompson Z, et al Glial Scar Borders Are Formed by Newly Proliferated, Elongated Astrocytes That Interact to Corral Inflammatory and Fibrotic Cells via STAT3-Dependent Mechanisms after Spinal Cord Injury. J Neurosci. 2013;33: 12870–12886. 10.1523/JNEUROSCI.2121-13.2013 23904622 PMC3728693

[16] Wilhelmsson U, Bushong EA, Price DL, Smarr BL, Phung V, Terada M, et al Redefining the concept of reactive astrocytes as cells that remain within their unique domains upon reaction to injury. Proc Natl Acad Sci U S A. 2006;103: 17513–17518. 17090684 10.1073/pnas.0602841103 PMC1859960

[17] Andrews EM, Richards RJ, Yin FQ, Viapiano MS, Jakeman LB. Alterations in chondroitin sulfate proteoglycan expression occur both at and far from the site of spinal contusion injury. Exp Neurol. 2012;235: 174–187. 10.1016/j.expneurol.2011.09.008 21952042 PMC3640493

[18] Galtrey CM, Fawcett JW. The role of chondroitin sulfate proteoglycans in regeneration and plasticity in the central nervous system. Brain Res Rev. 2007;54: 1–18. 17222456 10.1016/j.brainresrev.2006.09.006

[19] Laabs TL, Wang H, Katagiri Y, McCann T, Fawcett JW, Geller HM. Inhibiting glycosaminoglycan chain polymerization decreases the inhibitory activity of astrocyte-derived chondroitin sulfate proteoglycans. J Neurosci. 2007;27: 14494–14501. 18160657 10.1523/JNEUROSCI.2807-07.2007 PMC6673453

[20] Massey JM, Hubscher CH, Wagoner MR, Decker JA, Amps J, Silver J, et al Chondroitinase ABC digestion of the perineuronal net promotes functional collateral sprouting in the cuneate nucleus after cervical spinal cord injury. J Neurosci. 2006;26: 4406–4414. 16624960 10.1523/JNEUROSCI.5467-05.2006 PMC6673998

[21] Massey JM, Amps J, Viapiano MS, Matthews RT, Wagoner MR, Whitaker CM, et al Increased chondroitin sulfate proteoglycan expression in denervated brainstem targets following spinal cord injury creates a barrier to axonal regeneration overcome by chondroitinase ABC and neurotrophin-3. Exp Neurol. 2008;209: 426–445. 17540369 10.1016/j.expneurol.2007.03.029 PMC2270474

[22] Zhang F, Clarke JD, Ferretti P. FGF-2 Up-regulation and proliferation of neural progenitors in the regenerating amphibian spinal cord in vivo. Dev Biol. 2000;225: 381–391. 10985857 10.1006/dbio.2000.9843

[23] Blaugrund E, Lavie V, Cohen I, Solomon A, Schreyer DJ, Schwartz M. Axonal regeneration is associated with glial migration: comparison between the injured optic nerves of fish and rats. J Comp Neurol. 1993;330: 105–112. 8468398 10.1002/cne.903300109

[24] Lurie DI, Pijak DS, Selzer ME. Structure of reticulospinal axon growth cones and their cellular environment during regeneration in the lamprey spinal cord. J Comp Neurol. 1994;344: 559–580. 7929892 10.1002/cne.903440406

[25] Becker T, Wullimann MF, Becker CG, Bernhardt RR, Schachner M. Axonal regrowth after spinal cord transection in adult zebrafish. J Comp Neurol. 1997;377: 577–595. 9007194 10.1002/(sici)1096-9861(19970127)377:4<577::aid-cne8>3.0.co;2-#

[26] Colello RJ, Alexander JK. Chapter 6—Electrical Fields: Their Nature and Influence on Biological Systems In: Morkoc H, editor. Advanced Semiconductor and Organic Nano-Techniques. San Diego: Academic Press; 2003 pp. 319.

[27] McCaig CD, Zhao M. Physiological electrical fields modify cell behaviour; BioEssays. Bioessays. 1997;19: 819-819-826. 10.1002/bies.950190912 9297973

[28] McCaig CD, Song B, Rajnicek AM. Electrical dimensions in cell science. J Cell Sci. 2009;122: 4267–4276. 10.1242/jcs.023564 19923270

[29] Moulin VJ, Dube J, Rochette-Drouin O, Levesque P, Gauvin R, Roberge CJ, et al Electric Potential Across Epidermis and Its Role During Wound Healing Can Be Studied by Using an Reconstructed Human Skin. Adv Wound Care (New Rochelle). 2012;1: 81–87. 24527285 10.1089/wound.2011.0318 PMC3839018

[30] Song B, Zhao M, Forrester J, McCaig C. Nerve regeneration and wound healing are stimulated and directed by an endogenous electrical field in vivo. J Cell Sci. 2004;117: 4681–4690. 15371524 10.1242/jcs.01341

[31] Cortese B, Palama IE, D'Amone S, Gigli G. Influence of electrotaxis on cell behaviour. Integr Biol (Camb). 2014;6: 817–830. 25058796 10.1039/c4ib00142g

[32] Jahanshahi A, Schonfeld LM, Lemmens E, Hendrix S, Temel Y. In vitro and in vivo neuronal electrotaxis: a potential mechanism for restoration? Mol Neurobiol. 2014;49: 1005–1016. 10.1007/s12035-013-8575-7 24243342

[33] Li Y, Weiss M, Yao L. Directed migration of embryonic stem cell-derived neural cells in an applied electric field. Stem Cell Rev. 2014;10: 653–662. 10.1007/s12015-014-9518-z 24804615 PMC4243511

[34] Lin F, Baldessari F, Gyenge CC, Sato T, Chambers RD, Santiago JG, et al Lymphocyte electrotaxis in vitro and in vivo. J Immunol. 2008;181: 2465–2471. 18684937 10.4049/jimmunol.181.4.2465 PMC2572691

[35] McKasson MJ, Huang L, Robinson KR. Chick embryonic Schwann cells migrate anodally in small electrical fields. Exp Neurol. 2008;211: 585–587. 10.1016/j.expneurol.2008.02.015 18396278 PMC2483403

[36] Trollinger DR, Isseroff RR, Nuccitelli R. Calcium channel blockers inhibit galvanotaxis in human keratinocytes. J Cell Physiol. 2002;193: 1–9. 12209874 10.1002/jcp.10144

[37] Zhao M, Agius-Fernandez A, Forrester JV, McCaig CD. Orientation and directed migration of cultured corneal epithelial cells in small electric fields are serum dependent. J Cell Sci. 1996;109 (Pt 6): 1405–1414. 8799828 10.1242/jcs.109.6.1405

[38] Armstrong PF, Brighton CT, Star AM. Capacitively coupled electrical stimulation of bovine growth plate chondrocytes grown in pellet form. J Orthop Res. 1988;6: 265–271. 2830391 10.1002/jor.1100060214

[39] Blackiston DJ, McLaughlin KA, Levin M. Bioelectric controls of cell proliferation: ion channels, membrane voltage and the cell cycle. Cell Cycle. 2009;8: 3527–3536. 19823012 10.4161/cc.8.21.9888 PMC2862582

[40] Song B, Zhao M, Forrester JV, McCaig CD. Electrical cues regulate the orientation and frequency of cell division and the rate of wound healing in vivo. Proc Natl Acad Sci U S A. 2002;99: 13577–13582. 12368473 10.1073/pnas.202235299 PMC129716

[41] Zhao M, Forrester JV, McCaig CD. A small, physiological electric field orients cell division. Proc Natl Acad Sci U S A. 1999;96: 4942–4946. 10220398 10.1073/pnas.96.9.4942 PMC21796

[42] Zhao M, Song B, Pu J, Wada T, Reid B, Tai G, et al Electrical signals control wound healing through phosphatidylinositol-3-OH kinase-gamma and PTEN. Nature. 2006;442: 457–460. 16871217 10.1038/nature04925

[43] Mie M, Endoh T, Yanagida Y, Kobatake E, Aizawa M. Induction of neural differentiation by electrically stimulated gene expression of NeuroD2. J Biotechnol. 2003;100: 231–238. 12443854 10.1016/s0168-1656(02)00284-5

[44] Sauer H, Rahimi G, Hescheler J, Wartenberg M. Effects of electrical fields on cardiomyocyte differentiation of embryonic stem cells. J Cell Biochem. 1999;75: 710–723. 10572253 10.1002/(sici)1097-4644(19991215)75:4<710::aid-jcb16>3.0.co;2-z

[45] Hinkle L, McCaig CD, Robinson KR. The direction of growth of differentiating neurones and myoblasts from frog embryos in an applied electric field. J Physiol. 1981;314: 121–135. 7310685 10.1113/jphysiol.1981.sp013695 PMC1249421

[46] Jaffe LF, Poo MM. Neurites grow faster towards the cathode than the anode in a steady field. J Exp Zool. 1979;209: 115–128. 490126 10.1002/jez.1402090114

[47] McCaig CD. Spinal neurite reabsorption and regrowth in vitro depend on the polarity of an applied electric field. Development. 1987;100: 31–41. 3652966 10.1242/dev.100.1.31

[48] Orida N, Feldman JD. Directional protrusive pseudopodial activity and motility in macrophages induced by extracellular electric fields. Cell Motil. 1982;2: 243–255. 6816471 10.1002/cm.970020305

[49] Rajnicek AM, Robinson KR, McCaig CD. The direction of neurite growth in a weak DC electric field depends on the substratum: contributions of adhesivity and net surface charge. Dev Biol. 1998;203: 412–423. 9808790 10.1006/dbio.1998.9039

[50] McCaig CD, Rajnicek AM, Song B, Zhao M. Controlling cell behavior electrically: current views and future potential. Physiol Rev. 2005;85: 943–978. 15987799 10.1152/physrev.00020.2004

[51] Cao L, Wei D, Reid B, Zhao S, Pu J, Pan T, et al Endogenous electric currents might guide rostral migration of neuroblasts. EMBO Rep. 2013;14: 184–190. 10.1038/embor.2012.215 23328740 PMC3596136

[52] Foulds IS, Barker AT. Human skin battery potentials and their possible role in wound healing. Br J Dermatol. 1983;109: 515–522. 6639877 10.1111/j.1365-2133.1983.tb07673.x

[53] Nuccitelli R, Nuccitelli P, Li C, Narsing S, Pariser DM, Lui K. The electric field near human skin wounds declines with age and provides a noninvasive indicator of wound healing. Wound Repair Regen. 2011;19: 645–655. 10.1111/j.1524-475X.2011.00723.x 22092802 PMC3228273

[54] Chiang MC, Cragoe EJ Jr, Vanable JW Jr. Intrinsic electric fields promote epithelization of wounds in the newt, Notophthalmus viridescens. Dev Biol. 1991;146: 377–385. 1864462 10.1016/0012-1606(91)90239-y

[55] Katz U, Scheffey C. The voltage-dependent chloride current conductance of toad skin is localized to mitochondria-rich cells. Biochim Biophys Acta. 1986;861: 480–482. 3768357 10.1016/0005-2736(86)90457-8

[56] Scheffey C, Katz U. Current flow measurements from the apical side of toad skin. A vibrating probe analysis. Prog Clin Biol Res. 1986;210: 213–219. 3960911

[57] Bassett CA, Becker RO. Generation of electric potentials by bone in response to mechanical stress. Science. 1962;137: 1063–1064. 13865637 10.1126/science.137.3535.1063

[58] Candia OA, Zadunaisky JA, Bajandas F. Electrical potential profile of the isolated frog cornea. Invest Ophthalmol. 1968;7: 405–415. 5663551

[59] Chiang M, Robinson KR, Vanable JW Jr. Electrical fields in the vicinity of epithelial wounds in the isolated bovine eye. Exp Eye Res. 1992;54: 999–1003. 1521590 10.1016/0014-4835(92)90164-n

[60] Mathias RT, Rae JL, Baldo GJ. Physiological properties of the normal lens. Physiol Rev. 1997;77: 21–50. 9016299 10.1152/physrev.1997.77.1.21

[61] Parmelee JT, Robinson KR, Patterson JW. Effects of calcium on the steady outward currents at the equator of the rat lens. Invest Ophthalmol Vis Sci. 1985;26: 1343–1348. 4044162

[62] Parmelee JT. Measurement of steady currents around the frog lens. Exp Eye Res. 1986;42: 433–441. 3487463 10.1016/0014-4835(86)90003-5

[63] Borgens RB, Jaffe LF, Cohen MJ. Large and persistent electrical currents enter the transected lamprey spinal cord. Proc Natl Acad Sci U S A. 1980;77: 1209–1213. 6928670 10.1073/pnas.77.2.1209 PMC348455

[64] Reid B, Nuccitelli R, Zhao M. Non-invasive measurement of bioelectric currents with a vibrating probe. Nat Protoc. 2007;2: 661–669. 17406628 10.1038/nprot.2007.91

[65] Reid B, Song B, Zhao M. Electric currents in Xenopus tadpole tail regeneration. Dev Biol. 2009;335: 198–207. 10.1016/j.ydbio.2009.08.028 19733557

[66] Altizer AM, Stewart SG, Albertson BK, Borgens RB. Skin flaps inhibit both the current of injury at the amputation surface and regeneration of that limb in newts. J Exp Zool. 2002;293: 467–477. 12486807 10.1002/jez.10141

[67] Borgens RB, Vanable JW Jr, Jaffe LF. Bioelectricity and regeneration: large currents leave the stumps of regenerating newt limbs. Proc Natl Acad Sci U S A. 1977;74: 4528–4532. 270701 10.1073/pnas.74.10.4528 PMC431978

[68] Borgens RB, Vanable JW Jr, Jaffe LF. Role of subdermal current shunts in the failure of frogs to regenerate. J Exp Zool. 1979;209: 49–56. 314968 10.1002/jez.1402090106

[69] Eltinge EM, Cragoe EJ Jr, Vanable JW Jr. Effects of amiloride analogues on adult Notophthalmus viridescens limb stump currents. Comp Biochem Physiol A Comp Physiol. 1986;84: 39–44. 2871974 10.1016/0300-9629(86)90039-3

[70] Tweedell KS. The urodele limb regeneration blastema: the cell potential. ScientificWorldJournal. 2010;10: 954–971. 10.1100/tsw.2010.115 20526526 PMC5763810

[71] Becker RO. The bioelectric factors in amphibian-limb regeneration. J Bone Joint Surg Am. 1961;43-A: 643–656. 14448529

[72] Borgens RB, Vanable JW Jr, Jaffe LF. Bioelectricity and regeneration. I. Initiation of frog limb regeneration by minute currents. J Exp Zool. 1977;200: 403–416. 301554 10.1002/jez.1402000310

[73] Borgens RB, Vanable JW Jr, Jaffe LF. Reduction of sodium dependent stump currents disturbs urodele limb regeneration. J Exp Zool. 1979;209: 377–386. 490133 10.1002/jez.1402090304

[74] Borgens RB, Roederer E, Cohen MJ. Enhanced spinal cord regeneration in lamprey by applied electric fields. Science. 1981;213: 611–617. 7256258 10.1126/science.7256258

[75] Borgens RB, Toombs JP, Breur G, Widmer WR, Waters D, Harbath AM, et al An imposed oscillating electrical field improves the recovery of function in neurologically complete paraplegic dogs. J Neurotrauma. 1999;16: 639–657. 10447075 10.1089/neu.1999.16.639

[76] Jenkins LS, Duerstock BS, Borgens RB. Reduction of the current of injury leaving the amputation inhibits limb regeneration in the red spotted newt. Dev Biol. 1996;178: 251–262. 8812127 10.1006/dbio.1996.0216

[77] Levin M. Large-scale biophysics: ion flows and regeneration. Trends Cell Biol. 2007;17: 261–270. 17498955 10.1016/j.tcb.2007.04.007

[78] Smith SD. Effects of electrode placement on stimulation of adult frog limb regeneration. Ann N Y Acad Sci. 1974;238: 500–507. 4548335 10.1111/j.1749-6632.1974.tb26816.x

[79] Stewart S, Rojas-Muñoz A, Belmonte JCI. Bioelectricity and epimorphic regeneration. Bioessays. 2007;29: 1133–1137. 17935197 10.1002/bies.20656

[80] Ud-Din S, Sebastian A, Giddings P, Colthurst J, Whiteside S, Morris J, et al Angiogenesis is induced and wound size is reduced by electrical stimulation in an acute wound healing model in human skin. PLoS One. 2015;10: e0124502 10.1371/journal.pone.0124502 25928356 PMC4415761

[81] Smith SD. Induction of partial limb regeneration in Rana pipiens by galvanic stimulation. Anat Rec. 1967;158: 89–97. 6033441 10.1002/ar.1091580110

[82] Song B, Gu Y, Pu J, Reid B, Zhao Z, Zhao M. Application of direct current electric fields to cells and tissues in vitro and modulation of wound electric field in vivo. Nat Protoc. 2007;2: 1479–1489. 17545984 10.1038/nprot.2007.205

[83] Wang ET, Zhao M. Regulation of tissue repair and regeneration by electric fields. Chin J Traumatol. 2010;13: 55–61. 20109370

[84] Barker AT, Jaffe LF, Vanable JW Jr. The glabrous epidermis of cavies contains a powerful battery. Am J Physiol. 1982;242: R358–66. 7065232 10.1152/ajpregu.1982.242.3.R358

[85] Spence DW, Pomeranz B. Surgical wound healing monitored repeatedly in vivo using electrical resistance of the epidermis. Physiol Meas. 1996;17: 57–69. 8724518 10.1088/0967-3334/17/2/001

[86] Sun YH, Reid B, Fontaine JH, Miller LA, Hyde DM, Mogilner A, et al Airway epithelial wounds in rhesus monkey generate ionic currents that guide cell migration to promote healing. J Appl Physiol (1985). 2011;111: 1031–1041. 21719726 10.1152/japplphysiol.00915.2010 PMC3774198

[87] Lois N, Reid B, Song B, Zhao M, Forrester J, McCaig C. Electric currents and lens regeneration in the rat. Exp Eye Res. 2010;90: 316–323. 10.1016/j.exer.2009.11.007 19931246

[88] Wang E, Reid B, Lois N, Forrester JV, McCaig CD, Zhao M. Electrical inhibition of lens epithelial cell proliferation: an additional factor in secondary cataract? FASEB J. 2005;19: 842–844. 15764648 10.1096/fj.04-2733fje PMC1459287

[89] Borgens RB. Endogenous ionic currents traverse intact and damaged bone. Science. 1984;225: 478–482. 6740320 10.1126/science.6740320

[90] Rubinacci A, De Ponti A, Shipley A, Samaja M, Karplus E, Jaffe LF. Bicarbonate dependence of ion current in damaged bone. Calcif Tissue Int. 1996;58: 423–428. 8661484 10.1007/BF02509442

[91] Illingworth CM, Barker AT. Measurement of electrical currents emerging during the regeneration of amputated finger tips in children. Clin Phys Physiol Meas. 1980;1: 87-87-89.

[92] Becker RO, Spadaro JA. Electrical stimulation of partial limb regeneration in mammals. Bull N Y Acad Med. 1972;48: 627–641. 4503923 PMC1806700

[93] Becker RO. Stimulation of partial limb regeneration in rats. Nature. 1972;235: 109–111. 4550399 10.1038/235109a0

[94] Matic M, Lazetic B, Poljacki M, Djuran V, Matic A, Gajinov Z. Influence of different types of electromagnetic fields on skin reparatory processes in experimental animals. Lasers Med Sci. 2009;24: 321–327. 10.1007/s10103-008-0564-0 18536960

[95] Cao L, Pu J, Scott RH, Ching J, McCaig CD. Physiological electrical signals promote chain migration of neuroblasts by up-regulating P2Y1 purinergic receptors and enhancing cell adhesion. Stem Cell Rev. 2015;11: 75–86. 10.1007/s12015-014-9524-1 25096637 PMC4333314

[96] Chan CY, Nicholson C. Modulation by applied electric fields of Purkinje and stellate cell activity in the isolated turtle cerebellum. J Physiol. 1986;371: 89–114. 3701658 10.1113/jphysiol.1986.sp015963 PMC1192712

[97] Khan T, Myklebust J, Swiontek T, Sayers S, Dauzvardis M. Electrical field distribution within the injured cat spinal cord: injury potentials and field distribution. J Neurotrauma. 1994;11: 699–710. 7723069 10.1089/neu.1994.11.699

[98] Zuberi M, Liu-Snyder P, Ul Haque A, Porterfield DM, Borgens RB. Large naturally-produced electric currents and voltage traverse damaged mammalian spinal cord. J Biol Eng. 2008;2: 17-1611-2-17. 10.1186/1754-1611-2-17 19116024 PMC2647896

[99] Haan N, Song B. Therapeutic Application of Electric Fields in the Injured Nervous System. Adv Wound Care (New Rochelle). 2014;3: 156–165. 24761356 10.1089/wound.2013.0450 PMC3929243

[100] Levin M. Endogenous bioelectrical networks store non-genetic patterning information during development and regeneration. J Physiol. 2014;592: 2295–2305. 10.1113/jphysiol.2014.271940 24882814 PMC4048089

[101] Thompson DM, Koppes AN, Hardy JG, Schmidt CE. Electrical stimuli in the central nervous system microenvironment. Annu Rev Biomed Eng. 2014;16: 397–430. 10.1146/annurev-bioeng-121813-120655 25014787

[102] Zhao M. Electrical fields in wound healing-An overriding signal that directs cell migration. Semin Cell Dev Biol. 2009;20: 674–682. 10.1016/j.semcdb.2008.12.009 19146969

[103] Borgens RB, Blight AR, Murphy DJ, Stewart L. Transected dorsal column axons within the guinea pig spinal cord regenerate in the presence of an applied electric field. J Comp Neurol. 1986;250: 168–180. 3489013 10.1002/cne.902500204

[104] Borgens RB, Bohnert DM. The responses of mammalian spinal axons to an applied DC voltage gradient. Exp Neurol. 1997;145: 376–389. 9217074 10.1006/exnr.1997.6499

[105] Cork RJ, McGinnis ME, Tsai J, Robinson KR. The growth of PC12 neurites is biased towards the anode of an applied electrical field. J Neurobiol. 1994;25: 1509–1516. 7861115 10.1002/neu.480251204

[106] Erskine L, Stewart R, McCaig CD. Electric field-directed growth and branching of cultured frog nerves: effects of aminoglycosides and polycations. J Neurobiol. 1995;26: 523–536. 7602316 10.1002/neu.480260406

[107] Erskine L, McCaig CD. Growth cone neurotransmitter receptor activation modulates electric field-guided nerve growth. Dev Biol. 1995;171: 330–339. 7556917 10.1006/dbio.1995.1285

[108] Erskine L, McCaig CD. Integrated interactions between chondroitin sulphate proteoglycans and weak dc electric fields regulate nerve growth cone guidance in vitro. J Cell Sci. 1997;110 (Pt 16): 1957–1965. 9296394 10.1242/jcs.110.16.1957

[109] Koppes AN, Zaccor NW, Rivet CJ, Williams LA, Piselli JM, Gilbert RJ, et al Neurite outgrowth on electrospun PLLA fibers is enhanced by exogenous electrical stimulation. J Neural Eng. 2014;11: 046002-2560/11/4/046002. Epub 2014 Jun 3. 10.1088/1741-2560/11/4/046002 PMC4873603 24891494

[110] McCaig CD, Rajnicek AM, Song B, Zhao M. Has electrical growth cone guidance found its potential? Trends Neurosci. 2002;25: 354–359. 12079763 10.1016/s0166-2236(02)02174-4

[111] Patel N, Poo MM. Orientation of neurite growth by extracellular electric fields. J Neurosci. 1982;2: 483–496. 6279799 10.1523/JNEUROSCI.02-04-00483.1982 PMC6564252

[112] Pelletier SJ, Lagace M, St-Amour I, Arsenault D, Cisbani G, Chabrat A, et al The morphological and molecular changes of brain cells exposed to direct current electric field stimulation. Int J Neuropsychopharmacol. 2014;18: 10.1093/ijnp/pyu090 25522422 PMC4376545

[113] Stewart R, Erskine L, McCaig CD. Calcium channel subtypes and intracellular calcium stores modulate electric field-stimulated and -oriented nerve growth. Dev Biol. 1995;171: 340–351. 7556918 10.1006/dbio.1995.1286

[114] Wood MD, Willits RK. Applied electric field enhances DRG neurite growth: influence of stimulation media, surface coating and growth supplements. J Neural Eng. 2009;6: 046003-2560/6/4/046003. Epub 2009 Jun 3. 10.1088/1741-2560/6/4/046003 19494423

[115] Alexander J, Fuss B, Colello R. Electric field-induced astrocyte alignment directs neurite outgrowth. 2006;2: 93–103. 10.1017/S1740925X0600010X 18458757 PMC2367316

[116] Borgens RB, Shi R, Mohr TJ, Jaeger CB. Mammalian cortical astrocytes align themselves in a physiological voltage gradient. Exp Neurol. 1994;128: 41–49. 8070523 10.1006/exnr.1994.1111

[117] Babona Pilipos R, Popovic M, Morshead C. A galvanotaxis assay for analysis of neural precursor cell migration kinetics in an externally applied direct current electric field. 2012 10.3791/4193 23093363 PMC3490317

[118] Meijering E, Dzyubachyk O, Smal I. Methods for cell and particle tracking. Methods Enzymol. 2012;504: 183–200. 10.1016/B978-0-12-391857-4.00009-4 22264535

[119] R Core Team. R: A Language and Environment for Statistical Computing. Vienna, Austria: R Foundation for Statistical Computing; 2014.

[120] Agostinelli C, Lund U. R package Circular: Circular Statistics (version 0.4–7). CA: Department of Environmental Sciences, Informatics and Statistics, Ca' Foscari University, Venice, Italy. UL: Department of Statistics, California Polytechnic State University, San Luis Obispo, California, USA; 2013.

[121] Wickham H. ggplot2: elegant graphics for data analysis: Springer New York; 2009.

[122] Grosjean P, Ibanez F. pastecs: Package for Analysis of Space-Time Ecological Series; 2014.

[123] Wickham and Hadley. Reshaping data with the reshape package. 2007;21.

[124] Hothorn T, Bretz F, Westfall P. Simultaneous Inference in General Parametric Models. 2008;50: 346–363. 10.1002/bimj.200810425 18481363

[125] RStudio. RStudio. 2012;0.98.953.

[126] Schindelin J, Arganda-Carreras I, Frise E, Kaynig V, Longair M, Pietzsch T, et al Fiji: an open-source platform for biological-image analysis. Nat Methods. 2012;9: 676–682. 10.1038/nmeth.2019 22743772 PMC3855844

[127] Ayres C, Bowlin GL, Henderson SC, Taylor L, Shultz J, Alexander J, et al Modulation of anisotropy in electrospun tissue-engineering scaffolds: Analysis of fiber alignment by the fast Fourier transform. Biomaterials. 2006;27: 5524–5534. 16859744 10.1016/j.biomaterials.2006.06.014 PMC2929953

[128] Ayres CE, Jha BS, Meredith H, Bowman JR, Bowlin GL, Henderson SC, et al Measuring fiber alignment in electrospun scaffolds: a user's guide to the 2D fast Fourier transform approach. J Biomater Sci Polym Ed. 2008;19: 603–621. 10.1163/156856208784089643 18419940

[129] Tonar Z, Nemecek S, Holota R, Kocova J, Treska V, Molacek J, et al Microscopic image analysis of elastin network in samples of normal, atherosclerotic and aneurysmatic abdominal aorta and its biomechanical implications. J Appl Biomed. 2003;1: 149–159.

[130] Mutterer J, Zinck E. Quick-and-clean article figures with FigureJ. J Microsc. 2013;252: 89–91. 10.1111/jmi.12069 23906423

[131] Karimi-Abdolrezaee S, Billakanti R. Reactive astrogliosis after spinal cord injury-beneficial and detrimental effects. Mol Neurobiol. 2012;46: 251–264. 10.1007/s12035-012-8287-4 22684804

[132] Gotz M, Huttner WB. The cell biology of neurogenesis. Nat Rev Mol Cell Biol. 2005;6: 777–788. 16314867 10.1038/nrm1739

[133] Babona-Pilipos R, Droujinine IA, Popovic MR, Morshead CM. Adult subependymal neural precursors, but not differentiated cells, undergo rapid cathodal migration in the presence of direct current electric fields. PLoS One. 2011;6: e23808 10.1371/journal.pone.0023808 21909360 PMC3166127

[134] Feng JF, Liu J, Zhang XZ, Zhang L, Jiang JY, Nolta J, et al Guided migration of neural stem cells derived from human embryonic stem cells by an electric field. Stem Cells. 2012;30: 349–355. 10.1002/stem.779 22076946 PMC4437764

[135] Liu J, Zhu B, Zhang G, Wang J, Tian W, Ju G, et al Electric signals regulate directional migration of ventral midbrain derived dopaminergic neural progenitor cells via Wnt/GSK3beta signaling. Exp Neurol. 2014. 10.1016/j.expneurol.2014.09.014 25265211

[136] Yao L, Shanley L, McCaig C, Zhao M. Small applied electric fields guide migration of hippocampal neurons. J Cell Physiol. 2008;216: 527–535. 10.1002/jcp.21431 18393356

[137] McGinnis ME, Vanable JW. Electrical Fields in *Notophthalmus viridescens* Limb Stumps. Dev Biol. 1986;116: 184–193.

[138] Hill AB. The Environment and Disease: Association Or Causation? Proc R Soc Med. 1965;58: 295–300. 14283879 10.1177/003591576505800503 PMC1898525

[139] Levin M. Molecular bioelectricity in developmental biology: new tools and recent discoveries: control of cell behavior and pattern formation by transmembrane potential gradients. Bioessays. 2012;34: 205–217. 10.1002/bies.201100136 22237730 PMC3430077

[140] Gross D, Loew LM, Webb WW. Optical imaging of cell membrane potential changes induced by applied electric fields. Biophys J. 1986;50: 339–348. 3741986 10.1016/S0006-3495(86)83467-1 PMC1329750

[141] Binggeli R, Weinstein RC. Membrane potentials and sodium channels: hypotheses for growth regulation and cancer formation based on changes in sodium channels and gap junctions. J Theor Biol. 1986;123: 377–401. 2443763 10.1016/s0022-5193(86)80209-0

[142] Fang KS, Ionides E, Oster G, Nuccitelli R, Isseroff RR. Epidermal growth factor receptor relocalization and kinase activity are necessary for directional migration of keratinocytes in DC electric fields. J Cell Sci. 1999;112 (Pt 12): 1967–1978. 10341215 10.1242/jcs.112.12.1967

[143] Poo M. In situ electrophoresis of membrane components. Annu Rev Biophys Bioeng. 1981;10: 245–276. 7020576 10.1146/annurev.bb.10.060181.001333

[144] Tai G, Reid B, Cao L, Zhao M. Electrotaxis and wound healing: experimental methods to study electric fields as a directional signal for cell migration. 2009;571: 77–97. 10.1007/978-1-60761-198-1_5 19763960

[145] Zhao M, Pu J, Forrester JV, McCaig CD. Membrane lipids, EGF receptors, and intracellular signals colocalize and are polarized in epithelial cells moving directionally in a physiological electric field. FASEB J. 2002;16: 857–859. 11967227 10.1096/fj.01-0811fje

[146] Cooper MS, Miller JP, Fraser SE. Electrophoretic repatterning of charged cytoplasmic molecules within tissues coupled by gap junctions by externally applied electric fields. Dev Biol. 1989;132: 179–188. 2917693 10.1016/0012-1606(89)90216-9

[147] Stanimirovic DB, Ball R, Mealing G, Morley P, Durkin JP. The role of intracellular calcium and protein kinase C in endothelin-stimulated proliferation of rat type I astrocytes. Glia. 1995;15: 119–130. 8567063 10.1002/glia.440150204

[148] Yao L, McCaig CD, Zhao M. Electrical signals polarize neuronal organelles, direct neuron migration, and orient cell division. Hippocampus. 2009;19: 855–868. 10.1002/hipo.20569 19280605

[149] Gnanaguru G, Bachay G, Biswas S, Pinzon-Duarte G, Hunter DD, Brunken WJ. Laminins containing the beta2 and gamma3 chains regulate astrocyte migration and angiogenesis in the retina. Development. 2013;140: 2050–2060. 10.1242/dev.087817 23571221 PMC3631977

[150] Ho KW, Lambert WS, Calkins DJ. Activation of the TRPV1 cation channel contributes to stress-induced astrocyte migration. Glia. 2014;62: 1435–1451. 10.1002/glia.22691 24838827 PMC4107153

[151] Etienne-Manneville S, Hall A. Integrin-mediated activation of Cdc42 controls cell polarity in migrating astrocytes through PKCzeta. Cell. 2001;106: 489–498. 11525734 10.1016/s0092-8674(01)00471-8

[152] Calderwood DA, Campbell ID, Critchley DR. Talins and kindlins: partners in integrin-mediated adhesion. Nat Rev Mol Cell Biol. 2013;14: 503–517. 10.1038/nrm3624 23860236 PMC4116690

[153] Liao M, Cao E, Julius D, Cheng Y. Structure of the TRPV1 ion channel determined by electron cryo-microscopy. Nature. 2013;504: 107–112. 10.1038/nature12822 24305160 PMC4078027

[154] Cone CD Jr. Electroosmotic interactions accompanying mitosis initation in sarcoma cells in vitro. Trans N Y Acad Sci. 1969;31: 404–427. 5257510 10.1111/j.2164-0947.1969.tb02926.x

[155] Cone CD Jr, Tongier M Jr. Control of somatic cell mitosis by simulated changes in the transmembrane potential level. Oncology. 1971;25: 168–182. 5148061 10.1159/000224567

[156] Cone CD J, Cone C. Induction of mitosis in mature neurons in central nervous system by sustained depolarization; Science. Science. 1976. 10.1126/science.56781 56781

[157] Stillwell EF, Cone CM, Cone CD Jr. Stimulation of DNA synthesis in CNS neurones by sustained depolarisation. Nat New Biol. 1973;246: 110–111. 4518935 10.1038/newbio246110a0

[158] Wang E, Yin Y, Zhao M, Forrester JV, McCaig CD. Physiological electric fields control the G1/S phase cell cycle checkpoint to inhibit endothelial cell proliferation. FASEB J. 2003;17: 458–460. 12551844 10.1096/fj.02-0510fje

[159] Pappalardo LW, Samad OA, Black JA, Waxman SG. Voltage-gated sodium channel Nav 1.5 contributes to astrogliosis in an in vitro model of glial injury via reverse Na+ /Ca2+ exchange. Glia. 2014;62: 1162–1175. 10.1002/glia.22671 24740847 PMC4060891

[160] Tseng AS, Beane WS, Lemire JM, Masi A, Levin M. Induction of vertebrate regeneration by a transient sodium current. J Neurosci. 2010;30: 13192–13200. 10.1523/JNEUROSCI.3315-10.2010 20881138 PMC2965411

[161] Cahoy JD, Emery B, Kaushal A, Foo LC, Zamanian JL, Christopherson KS, et al A transcriptome database for astrocytes, neurons, and oligodendrocytes: a new resource for understanding brain development and function. J Neurosci. 2008;28: 264–278. 10.1523/JNEUROSCI.4178-07.2008 18171944 PMC6671143

[162] Dimou L, Gotz M. Glial cells as progenitors and stem cells: new roles in the healthy and diseased brain. Physiol Rev. 2014;94: 709–737. 10.1152/physrev.00036.2013 24987003

[163] Lee-Liu D, Edwards-Faret G, Tapia VS, Larrain J. Spinal cord regeneration: lessons for mammals from non-mammalian vertebrates. Genesis. 2013;51: 529–544. 10.1002/dvg.22406 23760835

[164] Barres BA. A new role for glia: generation of neurons! Cell. 1999;97: 667–670. 10380916 10.1016/s0092-8674(00)80777-1

[165] Buffo A, Rite I, Tripathi P, Lepier A, Colak D, Horn AP, et al Origin and progeny of reactive gliosis: A source of multipotent cells in the injured brain. Proc Natl Acad Sci U S A. 2008;105: 3581–3586. 10.1073/pnas.0709002105 18299565 PMC2265175

[166] Garcia AD, Doan NB, Imura T, Bush TG, Sofroniew MV. GFAP-expressing progenitors are the principal source of constitutive neurogenesis in adult mouse forebrain. Nat Neurosci. 2004;7: 1233–1241. 15494728 10.1038/nn1340

[167] Doetsch F, Caille I, Lim DA, Garcia-Verdugo JM, Alvarez-Buylla A. Subventricular zone astrocytes are neural stem cells in the adult mammalian brain. Cell. 1999;97: 703–716. 10380923 10.1016/s0092-8674(00)80783-7

[168] Doetsch F. The glial identity of neural stem cells. Nat Neurosci. 2003;6: 1127–1134. 14583753 10.1038/nn1144

[169] Lim DA, Tramontin AD, Trevejo JM, Herrera DG, Garca-Verdugo JM, Alvarez Buylla A. Noggin antagonizes BMP signaling to create a niche for adult neurogenesis. Neuron. 2000;28: 713–726. 11163261 10.1016/s0896-6273(00)00148-3

[170] Magnusson JP, Goritz C, Tatarishvili J, Dias DO, Smith EM, Lindvall O, et al A latent neurogenic program in astrocytes regulated by Notch signaling in the mouse. Science. 2014;346: 237–241. 10.1126/science.346.6206.237 25301628

[171] Miyata T, Kawaguchi A, Okano H, Ogawa M. Asymmetric inheritance of radial glial fibers by cortical neurons. Neuron. 2001;31: 727–741. 11567613 10.1016/s0896-6273(01)00420-2

[172] Ariza CA, Fleury AT, Tormos CJ, Petruk V, Chawla S, Oh J, et al The influence of electric fields on hippocampal neural progenitor cells. Stem Cell Rev. 2010;6: 585–600. 10.1007/s12015-010-9171-0 20665129

[173] Shi R, Borgens RB. Embryonic neuroepithelial sodium transport, the resulting physiological potential, and cranial development. Dev Biol. 1994;165: 105–116. 8088429 10.1006/dbio.1994.1238

