# Supplementary figures and images for: Elucidating the Role of Injury-Induced Electric Fields (EFs) in Regulating the Astrocytic Response to Injury in the Mammalian Central Nervous System
Source: PLoS One. 2015 Nov 12;10(11):e0142740. doi: 10.1371/journal.pone.0142740 (PMC4643040; doi:10.1371/journal.pone.0142740)

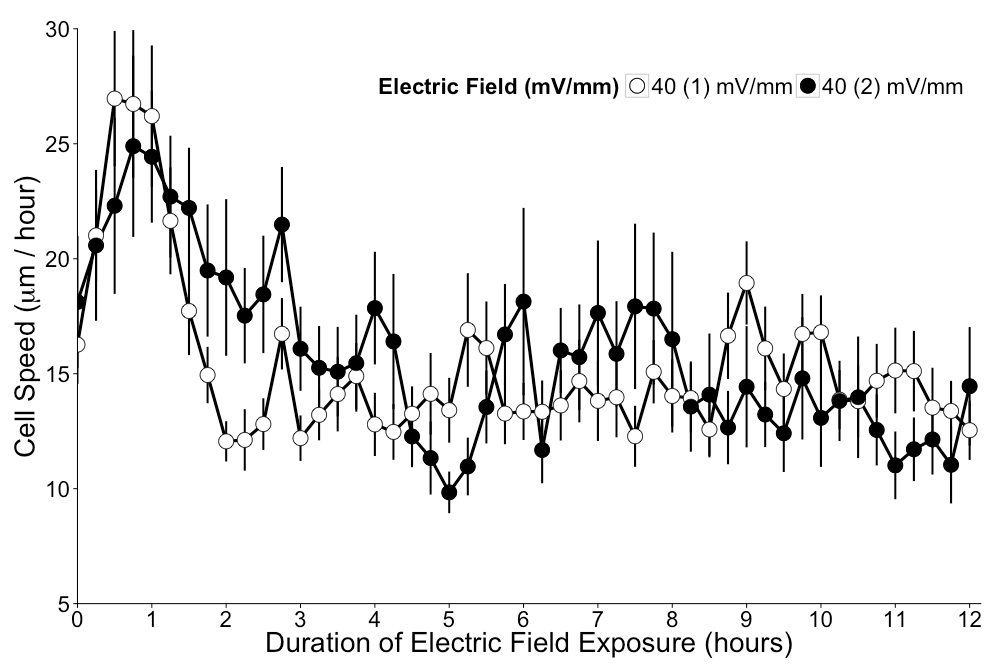

Supplement: S1 Fig — Sister cultures of astrocytes were exposed to a 40 mV/mm EF for 12 hours, with the EF exposure beginning either 16 (labeled 40 mV/mm (1)) or 48 (labeled 40 mV/mm (2)) hours after the cells were sub-cultured. No difference in the mean migration speed between these groups was found, indicating that the sequence in which astrocytes are exposed to each EF within an experiment does not serve as a confounding variable in these studies. Data are plotted as mean ± SEM. (TIF) [file pone.0142740.s005.tif]

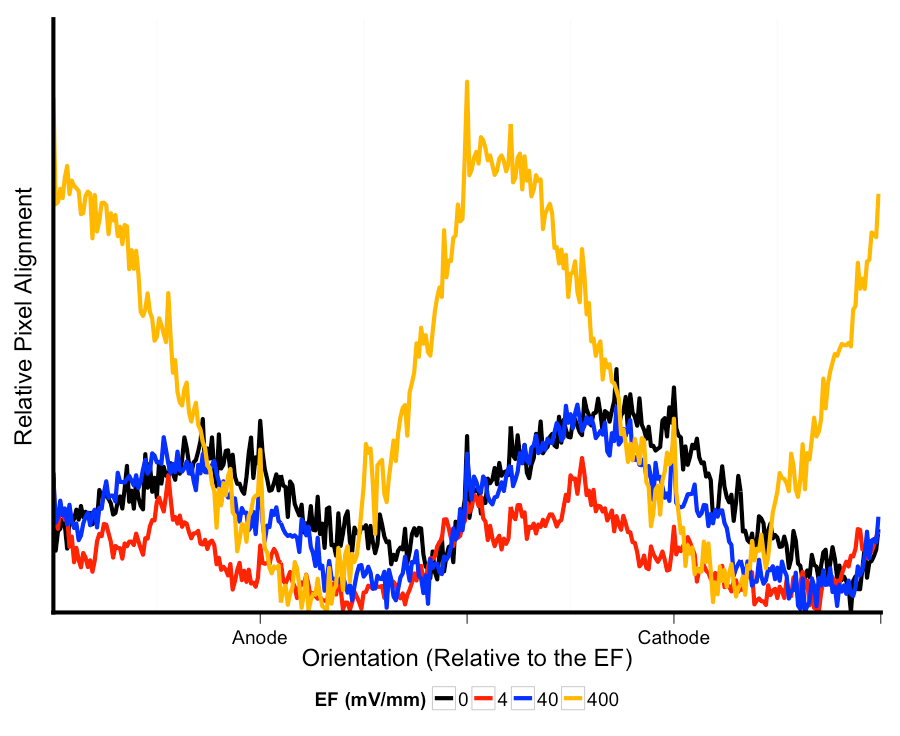

Supplement: S2 Fig — FFT analysis of normalized pixel intensity from vimentin immunolabeled images (averaged over 6–8 images) plotted as a function of direction relative to the anode and cathode. The high peaks for astrocytes exposed to 400 mV/mm demonstrate that astrocytes preferentially align their processes perpendicularly to the EF vector, while the absence of peaks for astrocytes exposed to 0, 4, or 40 mV/mm demonstrates that their processes are not aligned. All FFT graphs are plotted with the same scale in arbitrary units on the vertical axis; the horizontal axis indicates directionality relative to the anode and cathode. (TIF) [file pone.0142740.s006.tif]
